# Exploration of the Shared Gene Signatures and Molecular Mechanisms Between Systemic Lupus Erythematosus and Pulmonary Arterial Hypertension: Evidence From Transcriptome Data

**DOI:** 10.3389/fimmu.2021.658341

**Published:** 2021-07-15

**Authors:** Menghui Yao, Chunyi Zhang, Congcong Gao, Qianqian Wang, Mengmeng Dai, Runzhi Yue, Wenbo Sun, Wenfang Liang, Zhaohui Zheng

**Affiliations:** Department of Rheumatology, The First Affiliated Hospital of Zhengzhou University, Zhengzhou, China

**Keywords:** systemic lupus erythematosus, pulmonary arterial hypertension, WGCNA, differential gene analysis, type I IFN, miRNAs–mRNAs

## Abstract

**Background:**

Systemic lupus erythematosus (SLE) is an autoimmune disease that can affect multiple systems. Pulmonary arterial hypertension (PAH) has a close linkage with SLE. However, the inter-relational mechanisms between them are still unclear. This article aimed to explore the shared gene signatures and potential molecular mechanisms in SLE and PAH.

**Methods:**

The microarray data of SLE and PAH in the Gene Expression Omnibus (GEO) database were downloaded. The Weighted Gene Co-Expression Network Analysis (WGCNA) was used to identify the co-expression modules related to SLE and PAH. The shared genes existing in the SLE and PAH were performed an enrichment analysis by ClueGO software, and their unique genes were also performed with biological processes analyses using the DAVID website. The results were validated in another cohort by differential gene analysis. Moreover, the common microRNAs (miRNAs) in SLE and PAH were obtained from the Human microRNA Disease Database (HMDD) and the target genes of whom were predicted through the miRTarbase. Finally, we constructed the common miRNAs–mRNAs network with the overlapped genes in target and shared genes.

**Results:**

Using WGCNA, four modules and one module were identified as the significant modules with SLE and PAH, respectively. A ClueGO enrichment analysis of shared genes reported that highly activated type I IFN response was a common feature in the pathophysiology of SLE and PAH. The results of differential analysis in another cohort were extremely similar to them. We also proposed a disease road model for the possible mechanism of PAH secondary to SLE according to the shared and unique gene signatures in SLE and PAH. The miRNA–mRNA network showed that hsa-miR-146a might regulate the shared IFN-induced genes, which might play an important role in PAH secondary to SLE.

**Conclusion:**

Our work firstly revealed the high IFN response in SLE patients might be a crucial susceptible factor for PAH and identified novel gene candidates that could be used as biomarkers or potential therapeutic targets.

## Introduction

Pulmonary arterial hypertension (PAH) is a rare and severe cardiopulmonary disease, which is caused by cellular proliferation and fibrosis of pulmonary arterioles and arteries, leading to a progressive rise of pulmonary vascular resistance (PVR) and pulmonary arterial pressure (PAP), eventually resulting in right heart failure and death (1). PAH could be classified as idiopathic PAH (IPAH) and disease-associated PAH (APAH), the latter included the congenital heart disease-pulmonary artery hypertension (CHD-PAH) and connective tissue disease-pulmonary artery hypertension (CTD-PAH) (2). Systemic lupus erythematosus (SLE) has become the connective tissue disease that is closest with PAH in Japan, Korea, and China (3). The prevalence of PAH in the SLE patients varied from 0.5 to 43% (4). However, its prevalence in the general population is about 15–50 cases per million individuals (2), far lower than that in SLE patients. There must be some predisposing factors in SLE patients, which make them more prone to PAH. It is well-known that SLE is a chronic autoimmune inflammatory disease characterized by the formation of circulating autoantibody (5). At present, some studies have been demonstrated that some autoantibodies in SLE patients are highly related to PAH, such as anti-dsDNA antibody, anti-RNP antibody and antiphospholipid antibody (6, 7). However, these studies were mainly from serological perspectives, and fail to reveal the mechanism of PAH secondary to SLE at the genetic level.

With the quick development of gene microarray technology, researchers can measure the expression of thousands of genes data rapidly in various diseases, which help people to understand the pathogenesis of diseases more deeply from the genetic level. Recently, one meta-analysis of blood gene expression profiling about PAH found that IPAH showed highly similar expression profiling with no differentially expressed genes compared to APAH, even after substantially relaxing selection stringency, which mean the PAH had a relatively independent pathological process (8). The role of diseases causing PAH is more like a “trigger point”, once triggered, it can accelerate the development of PAH. To find out the “trigger point”, we tried to use the weighted gene co-expression network analysis (WGCNA) to identify the gene clusters of correlating and connected shared genes in SLE and PAH. This approach has been successfully applied in various biological contexts to identify common risk genes and mechanisms associated with multiple disease phenotypes (9–11).

Using the published gene expression data from the Gene Expression Omnibus (GEO) (http://www.ncbi.nlm.nih.gov/geo/), we identified the co-expression modules in SLE and PAH. Our analysis revealed the interferon (IFN)-induced genes were presented in modules highly related to SLE and PAH, and the biological pathway “type I IFN signaling pathway” might play salient role in PAH and SLE. Besides, the unique gene signatures in SLE and PAH were also identified. The results were confirmed in another cohort by differential gene analysis. To the best of our knowledge, this might be the first study to explore the shared gene signatures between SLE and PAH using a systems biology approach.

## Methods

### GEO Dataset Download and Process

We used the key word “system lupus erythematosus” or “pulmonary arterial hypertension” to search SLE and PAH gene expression profiles in the GEO database. The following criteria filter the obtained dataset: First, the gene expression profiling must include cases and controls. Second, the organization used for sequencing should be PBMC. Third, the number of samples in each group should not be less than 10 to ensure the accuracy of the WGCNA. Fourth, these datasets must provide the processed data or raw data that could be used for re-analyzation. Finally, the GEO dataset numbered GSE50772, GSE81622, GSE131793 and GSE703 were selected. The Series Matrix Files provided by the contributors include the normalized data processed by MAS5 algorithm. We then performed log2 transformed for gene expression profiling and matched the probes to their gene symbols according to the annotation document of corresponding platforms. Finally, the gene matrix with row names as sample names and column names as gene symbols were obtained for subsequent analyses.

### Weighted Gene Co-Expression Network Analysis

The weighted gene co-expression network analysis (WGCNA) is an algorithm that can find the co-expressed gene modules with high biological significance and explore the relationship between gene networks and diseases (12). Therefore, we used the WGCNA to obtain the SLE and PAH associated modules. More than 20,000 genes were obtained by sequencing in the GEO dataset, and most of these genes did not have different expression between samples, so we selected the first 25% of the genes with large variation according to their variance, including about 5,000 genes, for the WGCNA analysis. The “WGCNA” package in R.4.0.3 software were used to performed the WGCNA analysis. Before analysis, the hierarchical clustering analysis was performed using the Hclust function in R language to exclude the outlier samples. Then the appropriate soft powers β (ranged from 1 to 20) was selected using the function of “pickSoftThreshold” in the WGCNA package according to the standard of scale-free network. Next, the soft power value β and gene correlations matrix among all gene pairs calculated by Pearson analysis were used to build adjacency matrix, which was calculated by the formula: a_ij_ = |S_ij_|^β^ (a_ij_: adjacency matrix between gene i and gene j, S_ij_: similarity matrix which is composed of Pearson correlation coefficients of all gene pairs, β: soft power value). Then the topological overlap matrix (TOM) and the corresponding dissimilarity (1 − TOM) was transformed from the adjacency matrix. A hierarchical clustering dendrogram was further built and similar gene expressions were divided into different modules. Finally, the expression profiles of each module were summarized by the module eigengene (ME) and the correlation between the ME and clinical features was calculated. Therefore, the modules with high correlation coefficient with clinical features were focused and the genes in these modules were selected for subsequent analyses. In this study, the soft threshold β was 2 in the WGCNA analysis of SLE and 3 in PAH. The other parameters were the following: networkType = “unsigned”, minModuleSize = 20, mergeCutHeight = 0.25 and deepSplit = 2.

### Identification of Shared and Unique Gene Signatures in SLE and PAH

We selected the modules that were highly relevant to SLE and PAH. The shared genes in modules positively associated with SLE and PAH were overlapped using Jvenn (13). ClueGO is a Cytoscape plug-in, which could categorize the non-redundant GO terms and visualize them as a functionally grouped network (14). To explore potential roles of these shared genes in SLE and PAH, a biological analysis of these shared genes was performed by ClueGO. The biological process of GO analysis was focused. The *p*-value <0.05 was considered significant.

The protein–protein interaction (PPI) network and cluster analysis used the “MCODE” algorithm with default parameters in Cytoscape software (version: 3.7.2) were performed to identify the unique gene signatures in SLE and PAH. Gene clusters with scores >5 were focused. The biological processes of these unique genes were enriched by the DAVID website.

### Validation of Shared and Unique Gene Signatures Through DEGs Analysis

We performed the DEGs analysis on additional SLE and PAH datasets (GSE81622 and GSE703) to validate the shared and unique genes in SLE and PAH. The R packages “limma” was used to identify the DEGs between the case and control group. The cutoff value was |log2(foldchange)| >0.589, *p*-value <0.05. Hierarchical clustering heat maps were used to reveal the expression patterns of these DEGs. The overlapped DEGs in SLE and PAH database were obtained using Jvenn (13). We used the same methods to perform functional enrichment analyses on the common DEGs and total DEGs in SLE and PAH. The results of the enrichment analyses in the discovery cohort and the validation cohort were compared to determine whether our analysis method was reliable.

### Identified the Common MicroRNAs in SLE and PAH

MicroRNAs (miRNAs), small non-coding RNAs, have been demonstrated to modulate gene expression by promoting mRNA degradation or inhibiting mRNA translation (15). Therefore, we further explore whether some miRNAs are regulating these risk genes in SLE and PAH. The Human microRNA Disease Database (HMDD) is a database that curated experiment-supported evidence for human miRNA and disease associations (16). We obtained the SLE-associated and PAH-associated miRNAs and took an intersection of them. Then we further identified the expression levels of these miRNAs in SLE and PAH based on published literature, and only miRNAs with the same disorder types were further analyzed. To explore miRNAs’ function, we used the mirPath v3.0 software in DIANA tool to perform GO BP analysis. The GO terms with *p*-values <0.01 were considered significant.

### The Common miRNAs-Target Genes Network Construction

miRTarbase is an experimentally validated miRNA-target interactions database (http://mirtarbase.mbc.nctu.edu.tw/php/index.php), which included 4,076 miRNAs and 23,054 target genes supported by experimental evidence (reporter assay, Western blot, microarray, or pSILAC) (17). The intersection of target genes of common miRNAs and shared genes in SLE and PAH were used to construct the miRNAs–mRNAs regulated network. Cytoscape software was used to visualize the network.

## Results

### GEO Information

According to the previously set criteria, the four GEO datasets numbered GSE50772, GSE81622, GSE131793 and GSE703 were selected. The information of the four datasets were summarized in **Table 1**, such as GSE number, detection platforms, samples, and types of RNA sources. We further paired the GSE50772 and GSE131793 as a discovery cohort for the WGCNA analysis, and paired GSE81622 and GSE703 as a validated cohort for the DEG analysis.

**Table 1:** Summary of those four GEO datasets involving SLE and PAH patients.

ID	GSE number	Platform	Samples	Source types	Disease	Group
1	GSE50772	GPL570	61 patients and 20 controls	PBMC	SLE	Discovery cohort
2	GSE131793	GPL6244	10 patients and 10 controls	PBMC	PAH	Discovery cohort
3	GSE81622	GPL10558	30 patients and 25 controls	PBMC	SLE	Validation cohort
4	GSE703	GPL80	14 patients and six controls	PBMC	PAH	Validation cohort

### Discovery Cohort: The Co-Expression Modules in SLE and PAH

**Figure 1:**
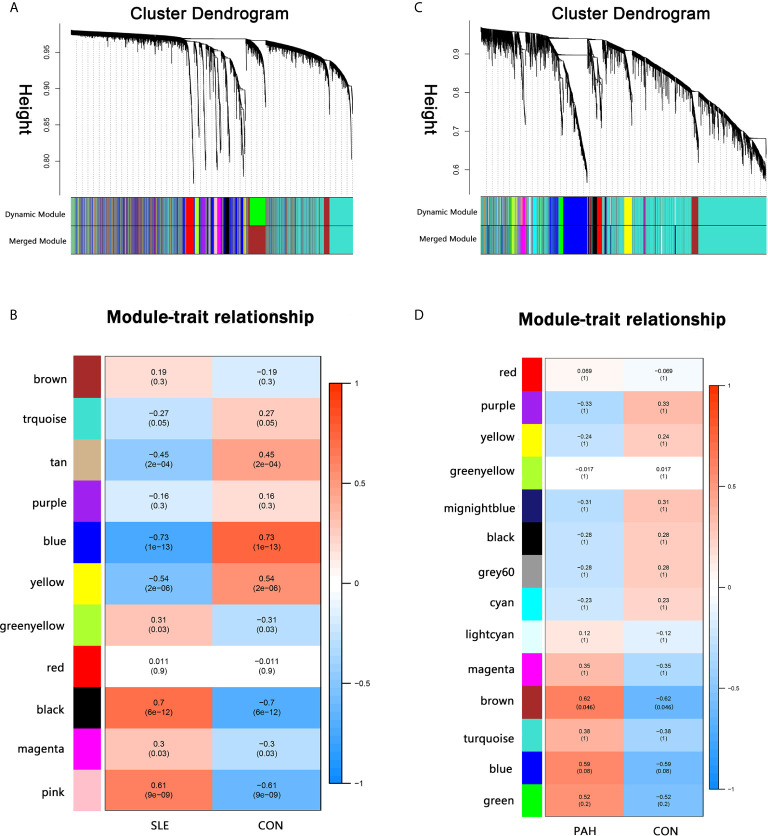
Weighted gene co-expression network analysis (WGCNA). **(A)** The cluster dendrogram of co-expression genes in SLE. **(B)** Module–trait relationships in SLE. Each cell contains the corresponding correlation and *p*-value. **(C)** The cluster dendrogram of co-expression genes in PAH. **(D)** Module–trait relationships in PAH. Each cell contains the corresponding correlation and *p*-value. SLE, systemic lupus erythematosus; PAH, pulmonary arterial hypertension.

A total of 11 modules were identified in GSE50772 through the WGCNA, with each color representing a different module. Then, a heat map was mapped about module–trait relationships according to the Spearman correlation coefficient to evaluate the association between each module and the disease (**Figures 1A, B**). Four modules “black”, “pink”, “yellow” and “blue” have high association with SLE and were selected as SLE-related modules (black module: r = 0.70, *p* = 6e−12; pink module: r = 0.61, p = 9e−09; blue module: r = −0.73, *p* = 1e−13, yellow module: r = −0.54, *p* = 2e−06). The black and pink modules were positively correlated with SLE, included 98 and 97 genes, respectively. The blue and yellow modules were negatively correlated with SLE, included 1,048 and 540 genes respectively. Similarly, a total of 14 modules were identified in GSE131793, and the module “brown” was the only one positively associated with PAH (r = 0.62, *p* = 0.046), including 415 genes (**Figures 1C, D**).

### The Common Gene Signatures in SLE and PAH

**Figure 2:**
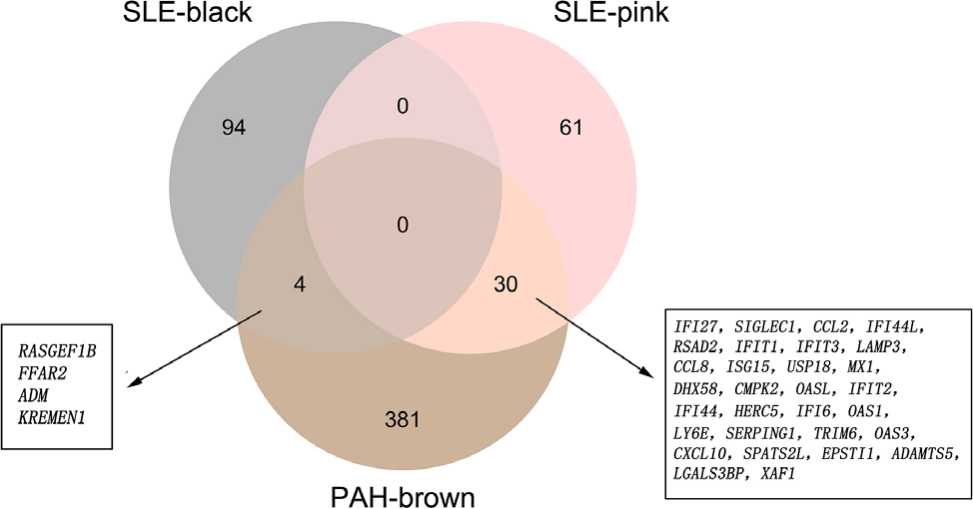
The shared genes between the black and pink modules of SLE and brown module of PAH by overlapping them. SLE, systemic lupus erythematosus; PAH, pulmonary arterial hypertension.

**Figure 3:**
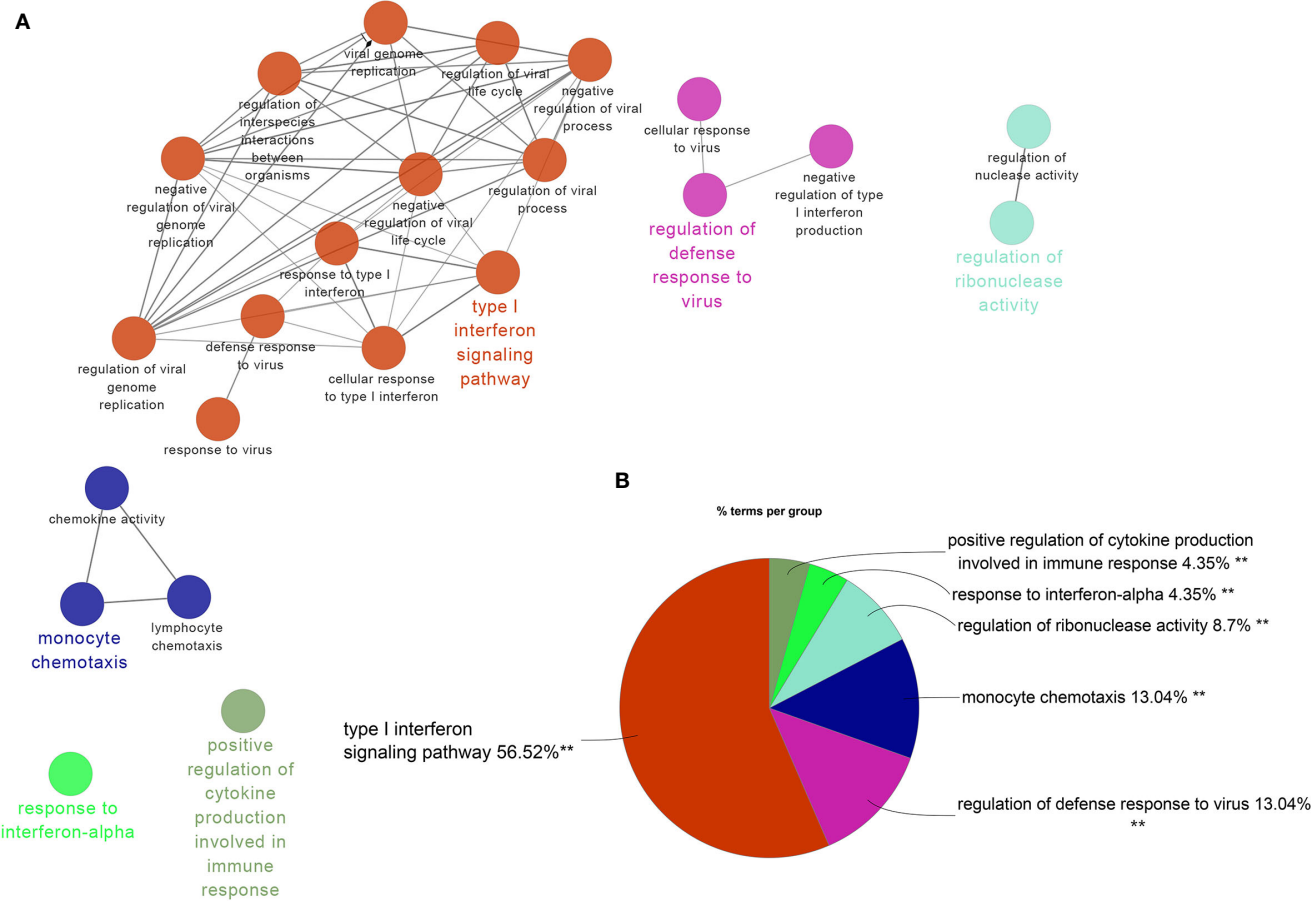
ClueGO enrichment analysis. **(A)** The interaction network of GO terms generated by the Cytoscape plug-in ClueGO. The significant term of each group is highlighted. **(B)** Proportion of each GO terms group in the total. GO, gene ontology. **p < 0.05.

There were 34 genes overlapped in positivity related modules of SLE and PAH, which was defined as gene set 1 (GS1) (**Figure 2**) and extremely related to the pathogenesis of SLE and PAH. To explore the potential functions of GS1, we performed the GO enrichment analysis with GlueGo. The top three significantly enriched GO terms about BP were “type I IFN signaling pathway”, “regulation of defense response to virus” and “monocyte chemotaxis”. Type I IFN signaling pathway accounted for 56% of total GO terms, and was associated with 22 genes (**Figures 3A, B**), confirming that this pathway maybe very important both in SLE and PAH.

### The Unique Gene Signatures in SLE and PAH

**Figure 4:**
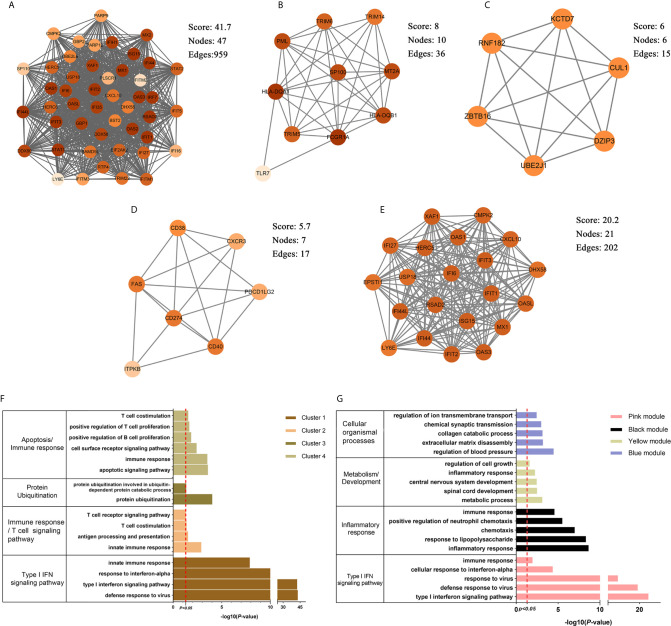
The PPI network and clusters analysis. **(A–D)** The clusters 1–4 extracted from brown module in PAH. **(E)** The cluster extracted from the pink module in SLE. **(F)** The GO biological process analyses of four gene clusters in PAH, one or two key words were used to summarize their main biological functions. **(G)** The GO biological process analyses of four genes modules in SLE, one or two key words were used to summarize their main biological functions. PPI, protein–protein network; SLE, systemic lupus erythematosus; PAH, pulmonary arterial hypertension; GO, gene ontology.

The brown module was the only one associated with PAH. We further constructed the PPI network at protein levels. Four clusters were extracted using MCODE analysis. Cluster 1 contained 47 nodes and 959 edges (score = 41.7). Functional enrichment analysis showed the genes of cluster 1 were mainly associated with type I IFN signaling pathways. Therefore, it was supposed that this gene cluster belonged to the gene part shared with SLE in PAH. The other three clusters were considered as unique gene signatures in PAH (**Figures 4A–D**). For each gene cluster, one or two keywords were chosen to summarize their main biological functions. Clusters 1 to 4 were mainly related to type I IFN signaling pathway, immune response/T cells signaling pathway, protein ubiquitination and apoptosis/immune response, respectively (**Figure 4F** and **Supplementary Tables 1–4**).

The pink, black, blue and yellow modules were closely associated with SLE, and the genes shared with PAH were mainly located in the pink module. Similarly, we constructed PPI network at the protein levels and used MCODE algorithm to extract the unique gene signature of SLE in pink module. However, only one cluster with 21 nodes and 202 edges score = 20.2) were extracted, and were mainly related to type I IFN signaling pathway (**Figure 4E**). Therefore, the pink module was considered to belong to the gene part shared with PAH in SLE. The other three modules were considered as unique gene signatures in SLE. The pink, black, blue and yellow module were mainly related to type I IFN signaling pathway, inflammatory response, metabolism/development and cellular organismal processes, respectively (**Figure 4G** and **Supplementary Tables 5–8**).

### Validated Cohort: The Differential Genes Analysis in SLE and PAH

**Figure 5:**
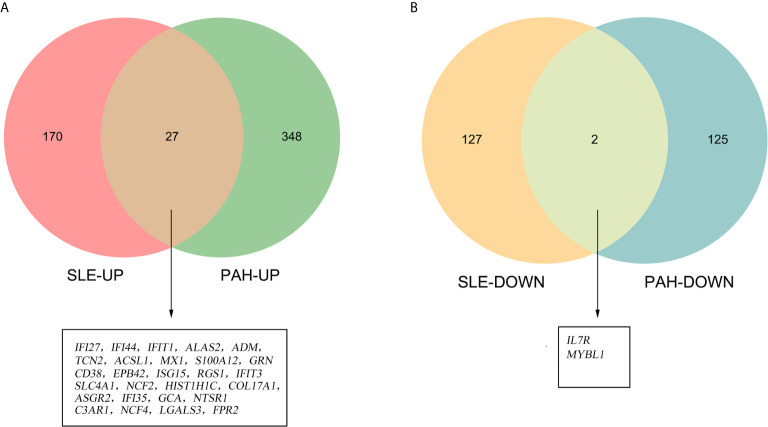
Identification of the common DEGs. **(A)** The Venn diagram of the upregulated genes in GSE81622 and GSE703. **(B)** The Venn diagram of the downregulated genes in GSE81622 and GSE703. DEGs differentially expressed genes.

**Figure 6:**
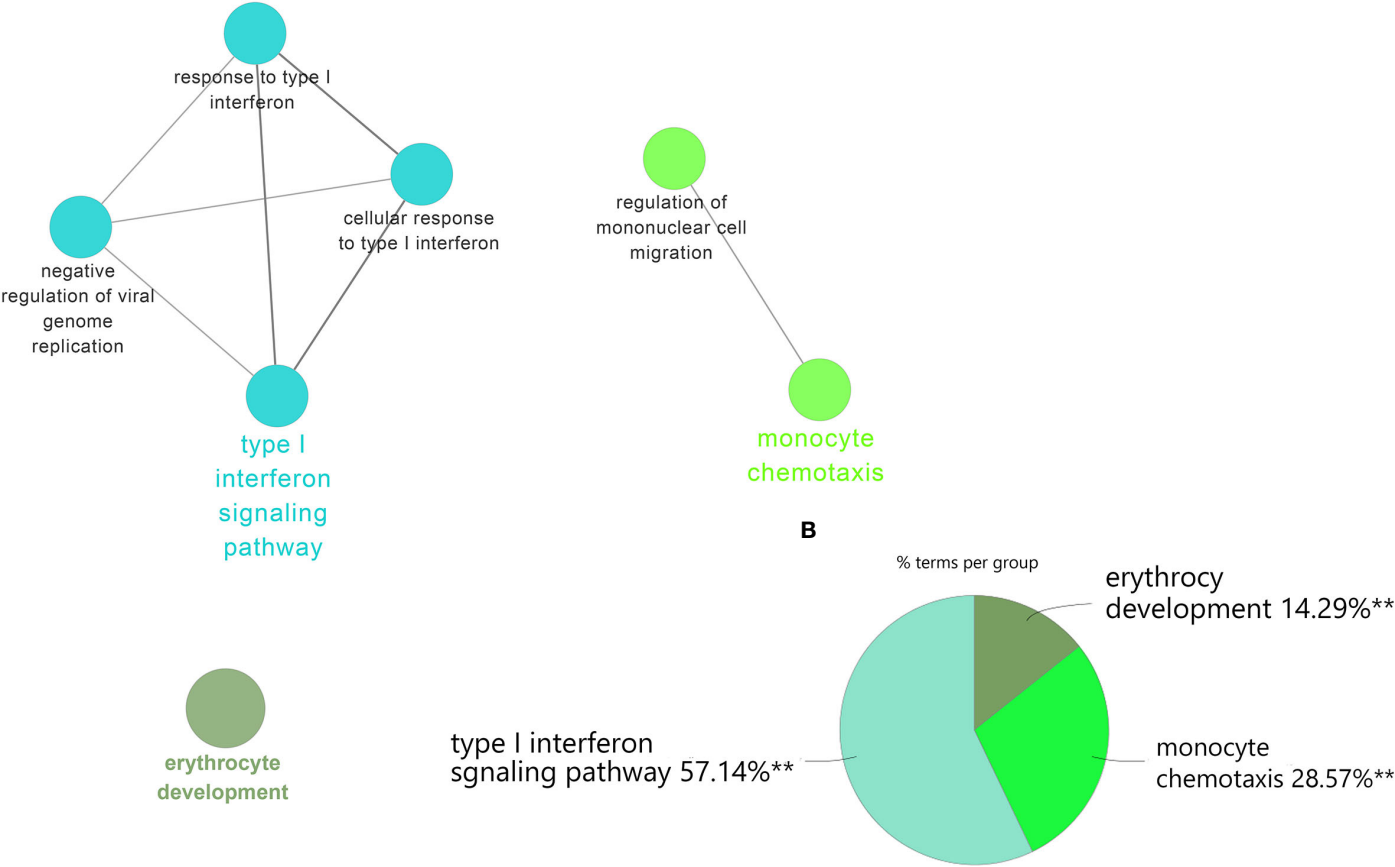
ClueGO enrichment analysis. **(A)** The interaction network of GO terms generated by the Cytoscape plug-in ClueGO. The significant term of each group is highlighted. **(B)** Proportion of each GO terms group in the total. GO, gene ontology. **p < 0.05.

To validate our results, we performed the differential genes analysis on the GSE81622 and GSE703 datasets. For GSE81622, a total of 326 DEGs were identified, including 197 upregulated genes and 129 downregulated genes. For GSE703, 502 DEGs were identified, including 375 upregulated genes and 127 downregulated genes. The hierarchical clustering suggested that the DEGs expression patterns were distinguishable between the case and control groups (**Supplementary Figure 1**). Among these DEGs, 27 genes were up-regulated and two genes were down-regulated in both GSE81622 and GSE703, which were defined as gene set 2 (GS2) (**Figure 5**). Functional enrichment analysis showed these DEGs were enriched in “type I interferon signaling pathway”, “monocyte chemotaxis” and “erythrocyte development”, which were consistent with those of the discovery cohort (**Figures 6A, B**). There are seven overlapped genes, *IFI27*, *IFI44*, *IFIT1*, *ADM*, *MX1*, *ISG15*, and *IFIT3* in GS1 and GS2, most of whom were IFN-induced genes.

We also performed the GO analyses on the DEGs in SLE and PAH. Besides type I IFN signaling pathway, inflammatory, immune response and multicellular organismal process including signaling transduction, cell migration, cell development and differentiation were also significantly enriched in SLE (**Supplementary Table 9**). For the DEGs in PAH, the apoptosis, immune response and T cells signaling pathway were also enriched, which was highly consistent with the results of the discovery cohort (**Supplementary Table 10**).

### Identified and Analysis of Common miRNAs in SLE and PAH

**Figure 7:**
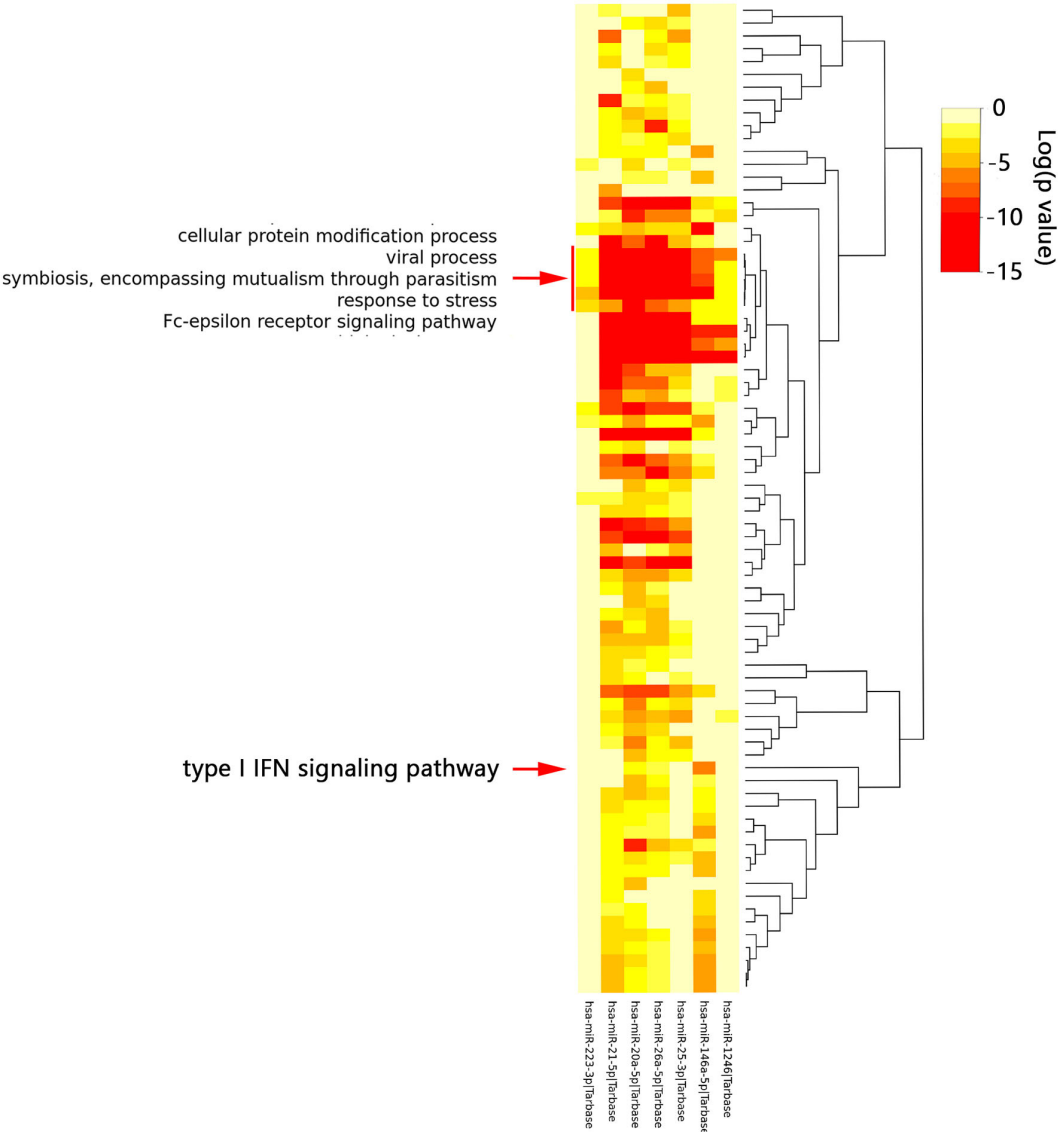
The functional enrichment analysis of seven common miRNAs. The red arrow indicated the type I interferon signaling pathway. The red box indicated the biological processes regulated by all seven miRNAs.

According to the HMDD database, 88 miRNAs were reported to be associated with SLE and 57 miRNAs were associated with PAH (**Supplementary Data 1**). There were 16 common miRNAs between SLE and PAH. According to the published literature provided by the HMDD database, we obtained the disorder types of these common miRNAs, there were six miRNAs (hsa-miR-1246, hsa-miR-146a-5p, hsa-miR-26a-5p, hsa-miR-25-3p, hsa-miR-223-3p and hsa-miR-20a-5p) downregulated and only hsa-miR-21-5p upregulated in both SLE and PAH. Then the seven miRNAs were further studied. The enrichment analysis showed that the functions of these miRNAs were involved in multiple biological processes. As shown in the heatmap, among these biological processes, the five biological processes, “Cellular protein modification process”, “Response to stress”, “Viral process”, “Fc-epsilon receptor signaling pathway”, and “Symbiosis, encompassing mutualism through parasitism” were regulated by all the seven miRNAs. Interestingly, the enrichment analysis also included the “type I IFN signaling pathway”, which indicated the common miRNAs involved in the pathogenesis of SLE and PAH could also regulate the type I IFN signaling pathway (**Figure 7** and **Supplementary Table 11**). That once again validated our analysis results.

### The Common miRNAs-Shared Genes Network

**Figure 8:**
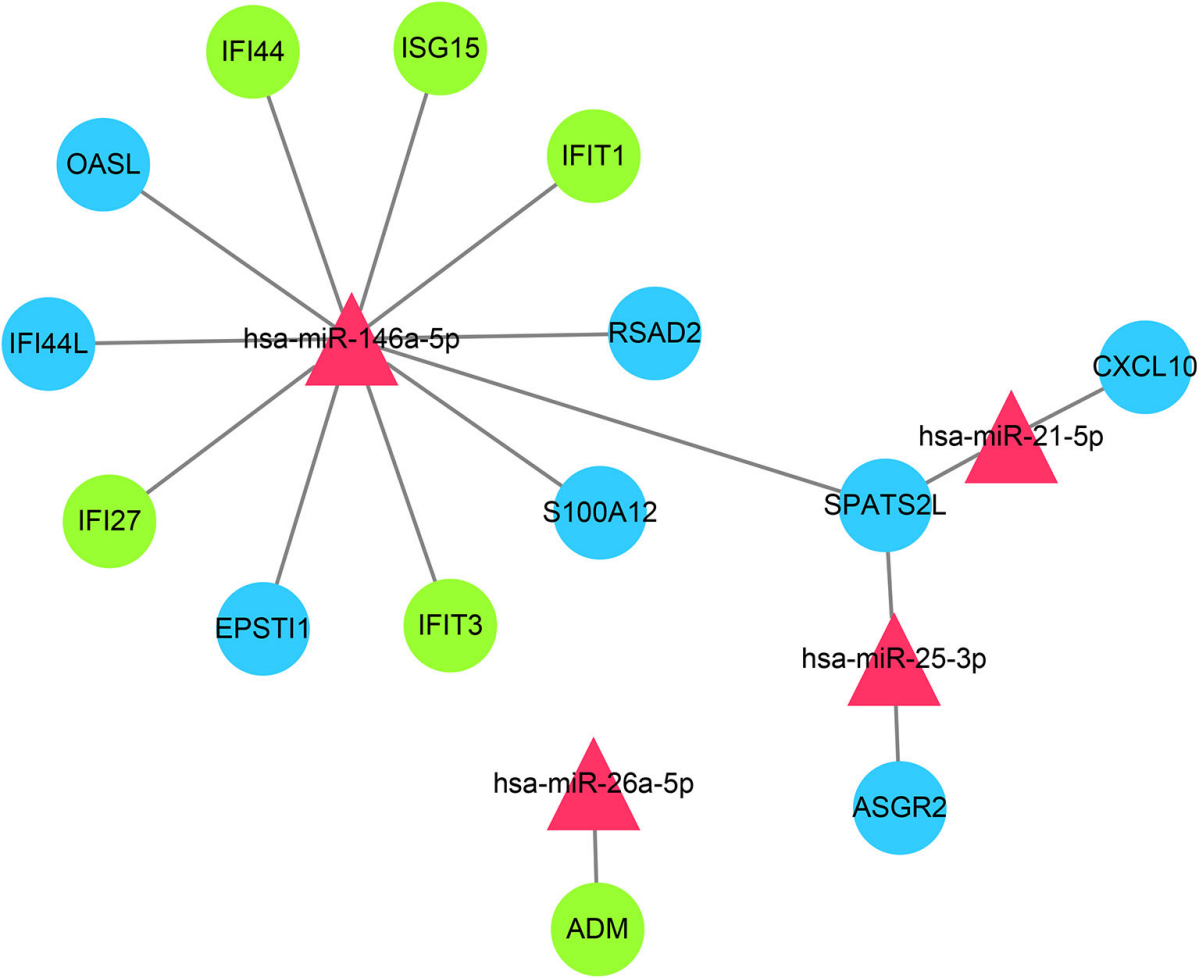
miRNAs–shared genes regulatory network. Red triangles represent miRNAs, and blue circles represent shared genes, green circles represent overlapped shared genes.

Through the miRTarbase, the 2,560 target genes of common miRNAs were predicted. Total fourteen genes in GS1 and GS2 were found in the 2,560 target genes, including six overlapped genes (*IFI27*, *IFI44*, *IFIT1*, *ADM*, *ISG15*, and *IFIT3*) in GS1 and GS2. Finally, the miRNAs–mRNAs network was constructed, including 18 nodes (four miRNAs, 14 mRNAs) and 16 edges (**Figure 8**). The IFN-induced genes were regulated by hsa-miR-146a. So, we hypothesized that the downregulated hsa-miR-146a might result in the upregulation of the IFN-induced genes in SLE and PAH.

## Discussion

The pathogenesis of SLE is still unclear yet, and the co-occurrence of SLE and PAH has been previously documented (6). Some serological biomarkers such as anti-dsDNA antibody, anti-SSA antibody, has been reported to be the risk factors for PAH development in SLE patients (18, 19). However, it seems that few studies have explored the susceptible aspects of PAH in SLE at the genetic level. For the first time, we explored the common mechanisms of SLE and PAH using the WGCNA, the algorithm can specially screen genes related to the clinical traits and obtain co-expression modules with high biological significance (12).

### Type I IFN, T Cell Activation, Apoptosis, and Protein Ubiquitination in PAH

Global gene expression studies of PBMC can help us better understand the specific pathobiology of PAH. The biological processes involved in the type I IFN response, immune response, apoptosis and protein ubiquitination were enriched among the co-expression gene modules in PAH. The highly active IFN response in the PAH gene signatures was very significant and consistent across multiple bioinformatic approaches. Previous studies have pointed out human pulmonary vascular cells sense type I IFN readily in the circulation and would release interferon γ inducible protein 10 (IP10) and endothelin-1 (ET-1) under the stimuli of high IFNα (20), which both play as key mediator in the pathogenesis of PAH (21, 22). Besides, the excessive IFN-induced genes were also found to result in the depletion of endothelial progenitor cells and the impairing of endothelial function, which may be related to the occurrence of PAH (23).

Some co-expressed genes associated with immune response and apoptosis were also identified in clusters 2 and 4 of the brown module. The T cell signaling pathway was repeatedly enriched in immune response. Therefore, the abnormal activation of T cells seemed to be significant in the pathogenesis of PAH. Recent studies showed the T help 17 (Th17), a subset of CD4^+^T cell, play an essential role in the initiation and maintenance of inflammation and promoted vascular remodeling, Th2 cells were involved in the muscularization of small pulmonary arteries. The excessive exhaustion of CD8^+^ T cells may have a close link to the imbalance of endothelial cells apoptotic–antiapoptotic signaling (24), which was consistent with our analysis. The genes for apoptosis and immune response were located in the same gene cluster.

The ubiquitin–proteasome system (UPS), as a major protein quality and quantity control system, regulates many cellular organic processes including signaling transduction, protein degradation, gene expression and apoptosis (25). Recent studies have reported that dysfunctions of UPS might be associated with the proliferation of pulmonary artery smooth muscle cells (SMC) in PAH (26), and some proteasome inhibitors, such as bortezomib and carfilzomib, have proven to be effective in treating PAH (27, 28).

Genes associated with these pathways were assigned to the one co-expression module, suggesting a positive correlation between gene clusters. Therefore, we speculated that these key pathways in PAH may interact and promote each other. Type I IFN could promote the proliferation, survival and differentiation of T cells through interferon α receptor (IFNAR1) and T cell receptor (TCR) (29). The over-activated T cells and excessive IFN-induced genes could also result in the endothelial cells’ apoptosis (24). Ubiquitination plays a broad role in various processes, some E3 ubiquitin ligases, including Cbl-b, c-Cbl, and Nrdp1, could regulate TCR-proximal signaling or conjugate non-degradative ubiquitin chain to Zap70 to regulate the activation of T cells (30), some E3 ubiquitin ligases, such as cIAP, could form distinct ubiquitin linkage chains to control the apoptosis (31). Therefore, the occurrence and development of PAH are very complicated. The high IFN response plays a critical role in this process. It forms a complex reciprocal network with many biological processes, including apoptosis, T cell activation, protein ubiquitination, etcetera.

### Type I IFN, Inflammatory Response, Cellular Organismal Processes in SLE

The activated type I IFN response and its associated autoimmune inflammation were well recognized as important features in SLE (32). Type I IFN was initially described and named for its ability to “interfere” with viral replication. Most studies demonstrated IFN also plays a role in regulating innate and adaptive immunity (33). Some viral infections, including the Epstein–Barr virus (EBV) and herpes simplex virus (HSV), have been thought to be associated with the onset of SLE (34). The abnormal immune complexes (ICs) containing viral nucleic acid encoded by EBV and other viruses could activate Toll-like receptors (TLRs) and their downstream pathways in plasmacytoid dendritic cells, stimulates IFNα production and T cell activation (34). Then T cells, B cells, pDCs and IFNα form a vicious circle, constantly producing autoantibodies and autoimmune cells (35). The cytokines, such as IL-1, IL-6, and TNFα, mediated by autoantibodies and ICs, would cause a persistent inflammatory response in SLE (36). Besides, a lot of evidence suggested an essential role for the neutrophil extracellular traps, also called NETosis, a particular form of death executed by neutrophils, in the pathogenesis of SLE (37).

However, the pathogenesis of SLE is exceptionally complicated. Besides the autoimmune inflammation mediated by IFNα, the blue and yellow modules related to aberrant multicellular organismal processes in PBMC of SLE were also identified by the WGCNA, including the metabolism, cell development, ion transport, signaling transduction, and so on. Zhang et al. performed a GO analysis of genes corresponding to differentially expressed proteins in SLE and also found cellular component organization, transport and multicellular organismal processes were significantly enriched, consistent with our analysis (38). For a long time, we focused on the IFN and autoimmune inflammation in SLE. However, the two main negative correlation modules with SLE suggested that the imbalance of homeostasis might also play an essential role in SLE.

### Disease Road Model—Type I IFN Acts as a Bridge From SLE to PAH

Multiple IPAH and APAH genome-wide blood expression studies have reported that there were no statistically significant differences in gene expression between APAH and IPAH (39, 40). One meta-analysis involving seven studies identified the DEGs in PAH, including APAH and IPAH, compared with healthy controls. The bioinformatics analysis showed high IFN response, antiviral response, autoimmune, cell death and T cell development were the unique gene signatures in PAH, which were consistent with our analyses (8). No difference between genetics suggested shared immunologic mechanisms in PAH. The presence of contributing diseases is more like a “trigger point”, triggering the onset of PAH.

As mentioned above, PAH has a high prevalence in SLE, suggesting that some susceptibility factors in SLE may trigger the onset and development of PAH. In our analyses, whether in the discovery cohort or the validation cohort, type I IFN played an extremely significant role in gene function enrichment analysis. Previous published studies are also similar to our findings. So, high IFN response may be a common feature in the pathophysiology of SLE and PAH. Interestingly, we also found immune response were enriched in both SLE and PAH, but we didn’t get the common genes associated immune response. In PAH, the immune-associated genes mainly included *CD274*, *CD40*, *CD38*, *TRIM5*, *TRIM6*, *TRIM14*, *HLA-DQA1*, *HLA-DQB1*, *FAS*, *PDCD1LG2*, and so on. Those genes were associated with T cell signaling pathway and apoptosis (41). However, the immune-associated genes in SLE were mainly associated with cytokines, including *IL1B*, *TNF*, *CXCL8*, *CCL20*, *CCL4*, *CXCL1*, *CXCL2*, *CXCL3*, *CXCR1*, *CXCR2* and so on. These indicated that the immune response of SLE was mainly related to the formation of an inflammatory environment, while the immune response in PAH was mainly related to T cell activation and immune-mediated apoptosis.

**Figure 9:**
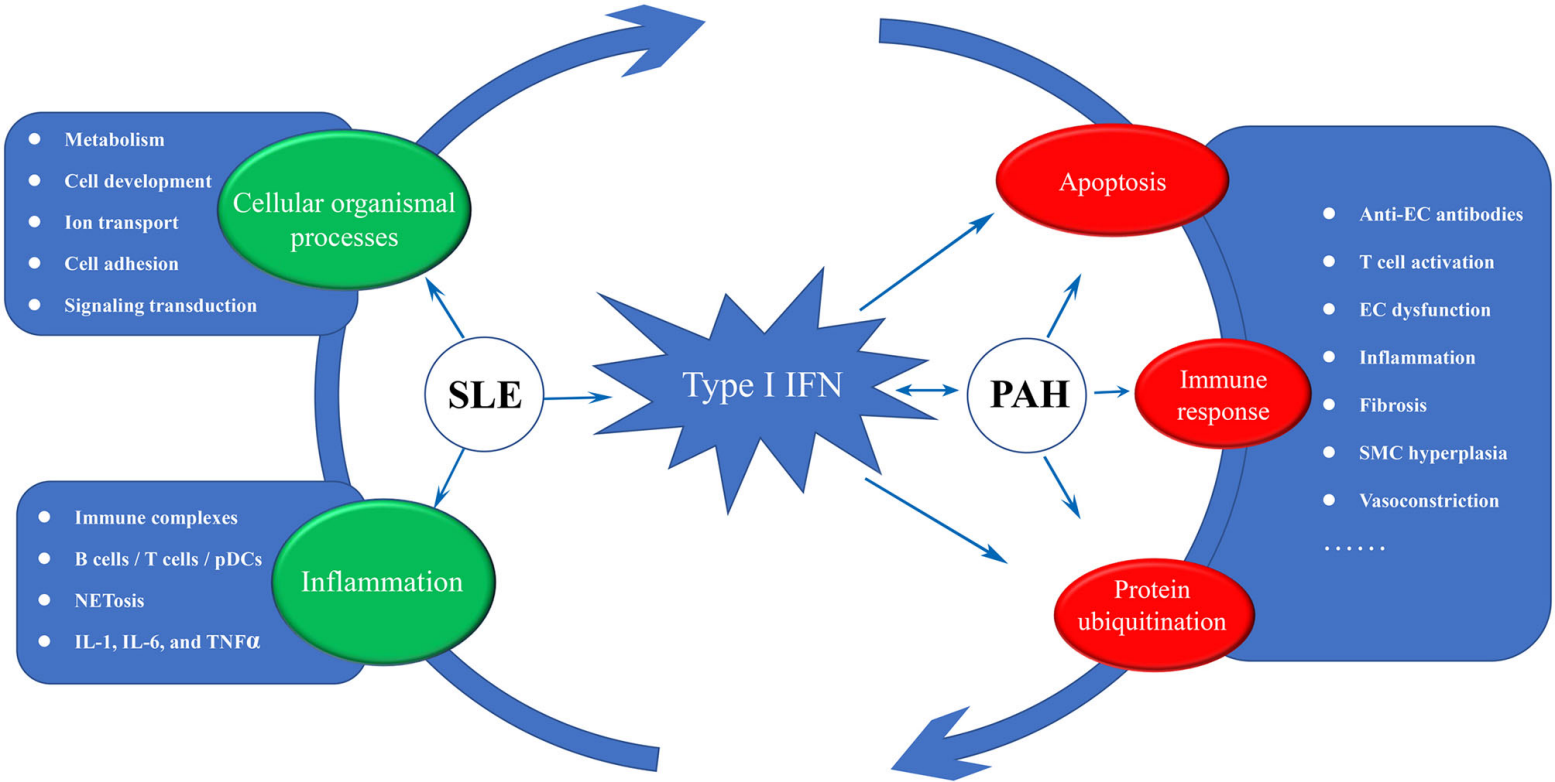
The disease road model. Type I IFN plays a central role in the disease road model. The impaired cellular organismal processes, persistent inflammation and high activated IFN response were significant characteristic in SLE, and some key words associated with three biological processes were display. In PAH, high IFN response forms a complex reciprocal network with the apoptosis, T cell activation and protein ubiquitination, resulting in inflammation and fibrosis. IFN, interferon; EC, endothelial cell; SMC, smooth muscle cell; SLE, systemic lupus erythematosus; PAH, pulmonary arterial hypertension.

Based on existing theories and our analysis results, we proposed a disease road model to illustrate the possible mechanism of PAH secondary to SLE. As shown in **Figure 9**, type I IFN plays a central role in the disease road model. In SLE, the autoantigens containing RNA or DNA could activate autoimmune B cells *via* B cell receptors (BCRs) and TLR, and promote them differentiate into plasma cells to release the autoantibodies (42). The abnormal ICs would cause continuous production of IFN and other inflammatory cytokines, such as IL-17, IL-1, and TNFα, *via* pDCs, B cells and T cells. In this process, a large number of cellular organismal processes were also damaged. The high IFN in the peripheral circulation of SLE patients could promote pulmonary vascular cells to release IP10 and ET-1, lead to an imbalance between vasoconstriction and vasodilatation factors. The direct damage of endothelial cells (EC) caused by IFN might trigger their apoptosis and generate anti-endothelial cell antibodies, and further activate the innate and adaptive immune, especially the abnormal activated T cell subsets, which resulted in the persistent inflammation and fibrosis. The activated UPS plays a catalytic role in the whole process. Eventually, under the comprehensive influence of the external environment, PAH were triggered.

### Emerging Therapeutic Target—Type I IFN and has-miR-146a

In recent years, despite the improvement in the management of the disease, the prognosis and survival rate of patients with SLE-PAH is still poor (43). The evidence from transcriptome data showed that IFN-induced genes played a central role in the pathophysiology of SLE and PAH, indicating anti-IFNα therapy may be an effective treatment for SLE–PAH. Some clinical studies have shown that the patients of SLE treated with anifrolumab, an anti-IFNα receptor antibody, had lower disease activity at multiple clinical endpoints than placebo (44). However, rontalizumab, another human monoclonal antibody against IFNα, did not show satisfactory results, the primary efficacy end point has not been achieved in this study (45). But the subgroup analyses showed positive effects in the group with a low IFN signature. Kalunian et al. thought the corresponding lack of response in patients with high disease activity might be due to low dose or complex multi-pathway in disease. From our analyses, inflammation response was a significant module juxtaposed with IFN module in SLE. Although there is a close relationship between inflammation and IFN, we think the inflammation in SLE was not necessarily caused by IFN. The influence of impaired cellular organismal processes on the treatment also needed to be further explored. Despite in fact that IFNα as a therapeutic intervention might fail, the potential protective effect of anti-IFNα therapy on target organs should be noticed. There was a complex reciprocal network, called cardiopulmonary–renal interaction (CPRI) involving maladaptive neurohormonal activation, abnormal immune signaling, oxidative stress, and cellular damage between heart, lung and kidney. The acute or chronic dysfunction of the lungs can result in clinically significant pathology in all three organs (46). Therefore, anti-IFNα therapy might have potential protective effect on lungs, heart and kidneys in SLE.

The function of post-transcriptional regulation of miRNA has been widely demonstrated to be associated the onset of various disease (15). Therefore, we took advantage of the HMDD database and the miRTarbase to construct the common miRNAs-shared genes network. It is interesting that the target genes of common miRNAs still included IFN-induced genes. Among these miRNAs, miR-146a had the most IFN-induced target genes. The miR-146a was significantly lower in SLE, and the frequency and expression level of “CC” genotype that was obviously associated with pulmonary hypertension were also decreased (47, 48). More importantly, Lu et al. found miR-146a prevalently expressed in Treg cell, and the deficiency of miR-146a in Treg cells would result in a breakdown of immunological tolerance (48, 49). With the function of negatively regulating the immune response and maintaining self-tolerances, Treg cells have proven to play a salient role in SLE and PAH (50). The downregulated expression of miR-146a in Treg cells resulted in the excessive activation of Stat1, and IFN-dependent immune-mediated organ damage would follow (49). Due to the critical role in regulating type IFN, miR-146a might be an important potential target for the treatment of SLE and PAH.

In conclusion, our work proposed a disease road model to illustrate the possible mechanism of PAH secondary to SLE, revealed the high IFN response in SLE might be an essential susceptible factor for PAH, and identifies novel gene candidates who could be used as biomarkers or as potential therapeutic targets.

## Data Availability Statement

The original contributions presented in the study are included in the article/**Supplementary Material**. Further inquiries can be directed to the corresponding author.

## Author Contributions

MY designed and conducted the whole research. CZ and CG applied for the GEO dataset analysis of SLE. MD and RY applied for the GEO dataset analysis of PAH. QW, WL, and WS were responsible for the analysis of miRNAs associated with SLE and PAH. MY completed the data analysis and drafted the manuscript. ZZ revised and finalized the manuscript. All authors contributed to the article and approved the submitted version.

## Funding

This work was supported by The Research Project of Medical Science and Technology of Henan Province of China [grant number LHGJ20190260] and The Key R & D Special Project of Henan Province of China [grant number 212102310754].

## Conflict of Interest

The authors declare that the research was conducted in the absence of any commercial or financial relationships that could be construed as a potential conflict of interest.

## Supplementary Material

Supplementary Figure 1 The differentially expressed analysis. **(A)** The heat map of the DEGs in GSE81622, red represents upregulation and green represents downregulation. **(B)** The heat map of the DEGs in GSE703, red represents upregulation and green represents downregulation. DEGs differentially expressed genes.
