# Supplementary figures and images for: Exploration of the Shared Gene Signatures and Molecular Mechanisms Between Systemic Lupus Erythematosus and Pulmonary Arterial Hypertension: Evidence From Transcriptome Data
Source: Front Immunol. 2021 Jul 15;12:658341. doi: 10.3389/fimmu.2021.658341 (PMC8320323; doi:10.3389/fimmu.2021.658341)

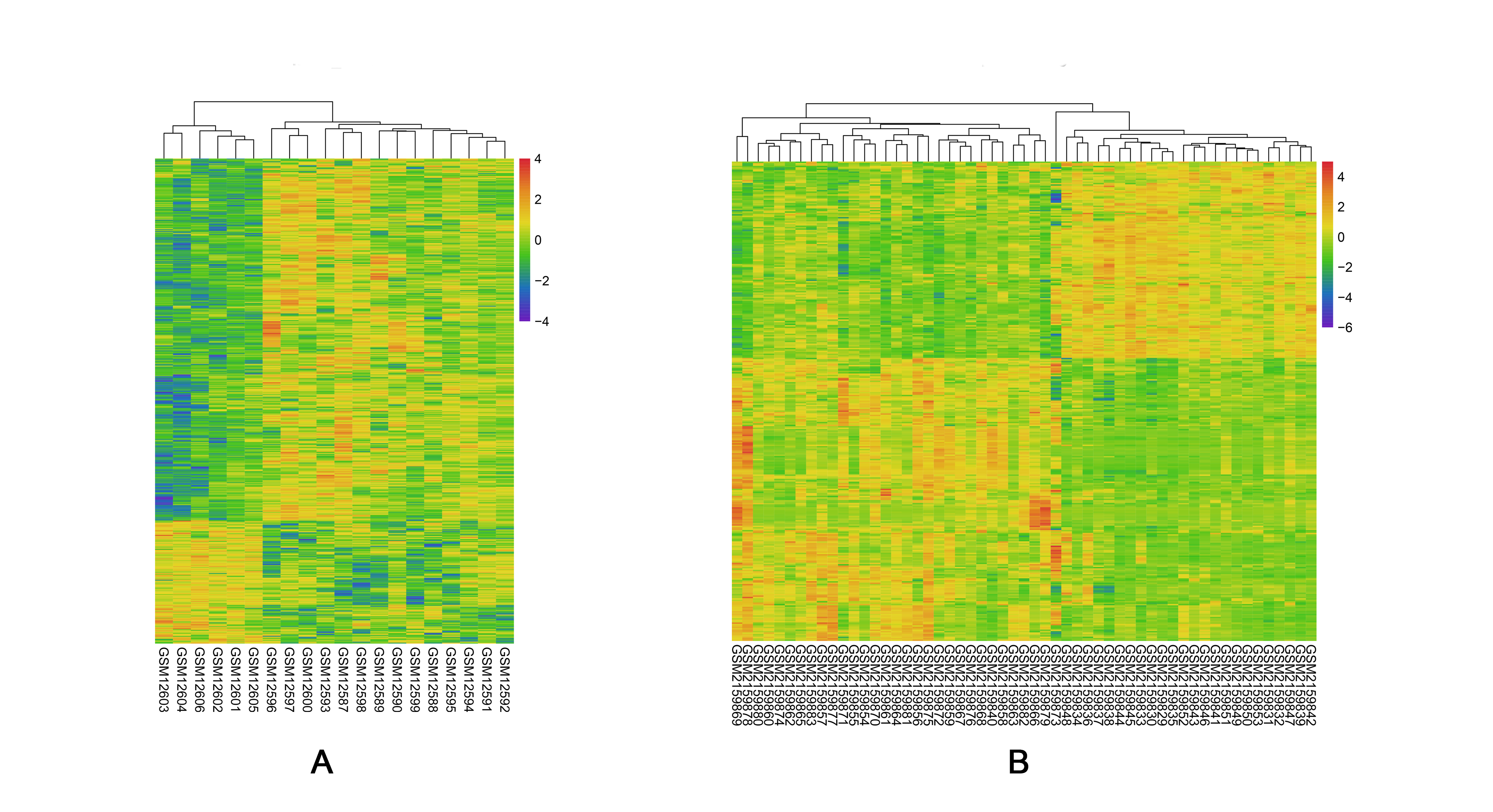

Supplement: Supplementary Figure 1 — The differentially expressed analysis. (A) The heat map of the DEGs in GSE81622, red represents upregulation and green represents downregulation. (B) The heat map of the DEGs in GSE703, red represents upregulation and green represents downregulation. DEGs differentially expressed genes. [file Image_1.jpeg]
