# Supplementary material for: Exploration of the Shared Gene Signatures and Molecular Mechanisms Between Systemic Lupus Erythematosus and Pulmonary Arterial Hypertension: Evidence From Transcriptome Data
Source: Front Immunol. 2021 Jul 15;12:658341. doi: 10.3389/fimmu.2021.658341 (PMC8320323; doi:10.3389/fimmu.2021.658341)
Supplement: Supplementary file 2 [file DataSheet_1.docx]

| GO numbers | GO terms | Counts | *P*-values | Genes |
| --- | --- | --- | --- | --- |
| GO:0051607 | defense response to virus | 28 | 9.90E-44 | *IFITM3, IFITM1, IFITM2, IFIT5, IFIT1, DDX60, IFI44L, IFIT3, IFIT2, OASL, HERC5, IFI16, DHX58, GBP1, TRIM22, RSAD2, STAT1, STAT2, MX2, MX1, EIF2AK2, ISG15, BST2, CXCL10, PLSCR1, OAS1, OAS2, OAS3* |
| GO:0060337 | type I interferon signaling pathway | 23 | 3.97E-43 | *IFITM3, IFITM1, IFITM2, RSAD2, STAT1, STAT2, MX2, MX1, IFI6, ISG15, IFI35, IFIT1, IFIT3, IFIT2, OASL, BST2, IFI27, OAS1, OAS2, OAS3, IRF7, GBP2, XAF1* |
| GO:0009615 | response to virus | 22 | 6.06E-35 | *IFITM3, IFITM1, IFITM2, RSAD2, DDX58, MX2, MX1, IFI44, EIF2AK2, IFIT1, DDX60, IFIT3, IFIT2, OASL, IFIH1, BST2, OAS1, OAS2, OAS3, DHX58, IRF7, TRIM22* |
| GO:0045071 | negative regulation of viral genome replication | 14 | 6.18E-25 | *IFITM3, IFITM1, IFITM2, RSAD2, MX1, EIF2AK2, ISG15, IFIT1, OASL, BST2, PLSCR1, IFI16, OAS1, OAS3* |
| GO:0035456 | response to interferon-beta | 7 | 2.18E-14 | *IFITM3, BST2, IFITM1, PLSCR1, IFITM2, STAT1, XAF1* |
| GO:0060333 | interferon-gamma-mediated signaling pathway | 9 | 1.29E-11 | *OAS1, STAT1, OAS2, OAS3, IRF7, GBP2, GBP1, TRIM22, OASL* |
| GO:0035455 | response to interferon-alpha | 6 | 2.74E-11 | *IFITM3, BST2, IFITM1, IFITM2, MX2, EIF2AK2* |
| GO:0045087 | innate immune response | 12 | 1.27E-08 | *IFIH1, HERC5, BST2, IFI16, DDX58, MX2, MX1, DHX58, IRF7, IFIT5, EIF2AK2, DDX60* |
| GO:0032480 | negative regulation of type I interferon production | 6 | 1.49E-08 | *IFIH1, HERC5, DDX58, DHX58, ISG15, UBE2L6* |
| GO:0006955 | immune response | 9 | 1.40E-05 | *IFITM3, CXCL10, IFITM2, OAS1, OAS2, OAS3, IFI6, GBP2, TRIM22* |

**Supplementary table 1: The GO biological process analysis of PAH cluster 1**

**Supplementary table 2: The GO biological process analysis of PAH cluster 2**

| GO numbers | GO terms | Counts | *P*-values | Genes |
| --- | --- | --- | --- | --- |
| GO:0045087 | innate immune response | 4 | 0.001249 | *TRIM5, TRIM14, TLR7, PML* |
| GO:0051091 | positive regulation of sequence-specific DNA binding transcription factor activity | 3 | 0.001355 | *SP100, TRIM5, TRIM14* |
| GO:0051607 | defense response to virus | 3 | 0.003302 | *TRIM5, TLR7, PML* |
| GO:0070206 | protein trimerization | 2 | 0.004815 | *TRIM6, TRIM5* |
| GO:0032897 | negative regulation of viral transcription | 2 | 0.006948 | *SP100, TRIM14* |
| GO:1902187 | negative regulation of viral release from host cell | 2 | 0.008545 | *TRIM5, PML* |
| GO:0002504 | antigen processing and presentation of peptide or polysaccharide antigen via MHC class II | 2 | 0.009077 | *HLA-DQA1, HLA-DQB1* |
| GO:0006955 | immune response | 3 | 0.02009 | *FCGR1A, HLA-DQA1, HLA-DQB1* |
| GO:0034097 | response to cytokine | 2 | 0.027534 | *SP100, PML* |
| GO:0019882 | antigen processing and presentation | 2 | 0.029102 | *HLA-DQA1, HLA-DQB1* |
| GO:0031295 | T cell costimulation | 2 | 0.041047 | *HLA-DQA1, HLA-DQB1* |
| GO:0019886 | antigen processing and presentation of exogenous peptide antigen via MHC class II | 2 | 0.048254 | *HLA-DQA1, HLA-DQB1* |
| GO:0050852 | T cell receptor signaling pathway | 2 | 0.048349 | *HLA-DQA1, HLA-DQB1* |

**Supplementary table 3: The GO biological process analysis of PAH cluster 3**

| GO numbers | GO terms | Counts | *P*-values | Genes |
| --- | --- | --- | --- | --- |
| GO:0016567 | protein ubiquitination | 4 | 9.39E-05 | *ZBTB16, RNF182, CUL1, UBE2J1* |
| GO:0042787 | protein ubiquitination involved in ubiquitin-dependent protein catabolic process | 2 | 0.04474 | *CUL1, DZIP3* |

**Supplementary table 4: The GO biological process analysis of PAH cluster 4**

| GO numbers | GO terms | Counts | *P*-values | Genes |
| --- | --- | --- | --- | --- |
| GO:0097190 | apoptotic signaling pathway | 3 | 2.62E-04 | *CD40, FAS, CD38* |
| GO:0006955 | immune response | 4 | 2.96E-04 | *CD274, CD40, FAS, PDCD1LG2* |
| GO:0046007 | negative regulation of activated T cell proliferation | 2 | 0.003212 | *CD274, PDCD1LG2* |
| GO:0007166 | cell surface receptor signaling pathway | 3 | 0.003811 | *ITPKB, CD274, CXCR3* |
| GO:0032693 | negative regulation of interleukin-10 production | 2 | 0.004281 | *CD274, PDCD1LG2* |
| GO:0007165 | signal transduction | 4 | 0.005626 | *ITPKB, CD274, FAS, CD38* |
| GO:0006954 | inflammatory response | 3 | 0.007177 | *CD40, CXCR3, FAS* |
| GO:0032689 | negative regulation of interferon-gamma production | 2 | 0.009965 | *CD274, PDCD1LG2* |
| GO:0030890 | positive regulation of B cell proliferation | 2 | 0.013857 | *CD40, CD38* |
| GO:0042102 | positive regulation of T cell proliferation | 2 | 0.021251 | *CD274, PDCD1LG2* |
| GO:0071260 | cellular response to mechanical stimulus | 2 | 0.025106 | *CD40, FAS* |
| GO:0031295 | T cell costimulation | 2 | 0.027553 | *CD274, PDCD1LG2* |
| GO:0006461 | protein complex assembly | 2 | 0.040745 | *CD40, FAS* |
| GO:0033209 | tumor necrosis factor-mediated signaling pathway | 2 | 0.041435 | *CD40, FAS* |
| GO:0007204 | positive regulation of cytosolic calcium ion concentration | 2 | 0.046942 | *CXCR3, CD38* |

**Supplementary table 5: The GO biological process analysis of pink module in SLE**

| GO numbers | GO terms | Counts | *P*-values | Genes |
| --- | --- | --- | --- | --- |
| GO:0060337 | type I interferon signaling pathway | 12 | 7.33E-23 | *IFI27, RSAD2, OAS3, MX1, IFI6, ISG15, XAF1, IFIT1, IFIT3, IFIT2, OASL, OAS1* |
| GO:0051607 | defense response to virus | 13 | 2.55E-20 | *RSAD2, MX1, ISG15, IFIT1, IFIT3, IFI44L, IFIT2, OASL, HERC5 CXCL10, OAS1, OAS3, DHX58* |
| GO:0009615 | response to virus | 10 | 1.38E-15 | *RSAD2, OAS1, OAS3 MX1, DHX58, IFI44, IFIT1, IFIT3, IFIT2, OASL* |
| GO:0045071 | negative regulation of viral genome replication | 7 | 3.27E-12 | *RSAD2, OAS1, OAS3, MX1, ISG15, IFIT1, OASL* |
| GO:0035457 | cellular response to interferon-alpha | 3 | 4.35E-05 | *OAS1, IFIT3, IFIT2* |
| GO:0032480 | negative regulation of type I interferon production | 3 | 5.18E-04 | *HERC5, DHX58, ISG15* |
| GO:0060700 | regulation of ribonuclease activity | 2 | 0.002262 | *OAS1, OAS3* |
| GO:0060333 | interferon-gamma-mediated signaling pathway | 3 | 0.002877 | *OAS1, OAS3, OASL* |
| GO:0032020 | ISG15-protein conjugation | 2 | 0.006771 | *HERC5, ISG15* |
| GO:0034340 | response to type I interferon | 2 | 0.007895 | *MX1, ISG15* |
| GO:0006955 | immune response | 4 | 0.011249 | *CXCL10, OAS1, OAS3, IFI6* |

**Supplementary table 6: The GO biological process analysis of black module in SLE**

| GO numbers | GO terms | Counts | *P*-values | Genes |
| --- | --- | --- | --- | --- |
| GO:0006954 | inflammatory response | 15 | 2.31E-09 | *CXCL8, TNFAIP6, CCL20, TNFRSF10C, CXCL1, FOS, CXCL3, PTGS2, CXCL2, TNF, CXCR1, IL1B, CCL4, CXCR2, PROK2* |
| GO:0032496 | response to lipopolysaccharide | 11 | 4.84E-09 | *JUN, GJB6, TNFRSF10C, ALPL, ADM, CXCL1, FOS, CXCL3, TRIB1, PTGS2, CXCL2* |
| GO:0070098 | chemokine-mediated signaling pathway | 8 | 4.06E-08 | *CXCL8, CXCR1, CCL20, CXCR2, CCL4, CXCL1, CXCL3, CXCL2* |
| GO:0006935 | chemotaxis | 9 | 1.04E-07 | *FOSL1, CXCL8, CXCR1, CCL20, CXCR2, PROK2, CXCL1, CMTM2, CXCL2* |
| GO:0090023 | positive regulation of neutrophil chemotaxis | 5 | 3.11E-06 | *CXCL8, CXCR2, CXCL1, CXCL3, CXCL2* |
| GO:0048661 | positive regulation of smooth muscle cell proliferation | 6 | 9.04E-06 | *JUN, NAMPT, PTGS2, TNF, EREG, HBEGF* |
| GO:0030593 | neutrophil chemotaxis | 6 | 1.45E-05 | *CXCL8, CCL20, IL1B, CXCR2, CCL4, CXCL3* |
| GO:0006955 | immune response | 11 | 2.71E-05 | *FCGR3B, CXCL8, CCL20, IL1B, CCL4, OSM, TNFRSF10C, CXCL1, CXCL3, TNF, CXCL2* |
| GO:0008285 | negative regulation of cell proliferation | 10 | 9.87E-05 | *FOSL1, JUN, CXCL8, IL1B, GJB6, OSM, ADM, CXCL1, PTGS2, EREG* |
| GO:0007267 | cell-cell signaling | 8 | 1.91E-04 | *NOV, TNFAIP6, CCL20, IL1B, NAMPT, CCL4, ADM, EREG* |
| GO:0042127 | regulation of cell proliferation | 7 | 2.35E-04 | *NFKBIA, JUN, TNFRSF10C, CXCL3, SGK1, TNF, CXCL2* |
| GO:0051384 | response to glucocorticoid | 5 | 2.47E-04 | *APOA2, ALPL, ADM, PTGS2, TNF* |
| GO:0060326 | cell chemotaxis | 5 | 2.47E-04 | *NOV, CCL20, CXCL1, CXCL2, HBEGF* |
| GO:0007565 | female pregnancy | 5 | 8.18E-04 | *FOSL1, NAMPT, FOSB, ADM, FOS* |
| GO:0045766 | positive regulation of angiogenesis | 5 | 0.002113 | *CXCL8, LRG1, IL1B, CXCR2, ADM* |
| GO:0019221 | cytokine-mediated signaling pathway | 5 | 0.003384 | *SOCS3, IL1B, LRRC4, IFNA8, EREG* |
| GO:0001525 | angiogenesis | 6 | 0.004013 | *JUN, CXCL8, NOV, PROK2, PTGS2, EREG* |
| GO:0008284 | positive regulation of cell proliferation | 8 | 0.006297 | *FOSL1, NAMPT, CXCR2, OSM, PROK2, ADM, EREG, HBEGF* |
| GO:0007165 | signal transduction | 13 | 0.007786 | *CXCL8, CD83, TNFAIP6, CCL20, TNFRSF10C, ADM, CXCL1, NR4A2, IL1B, CCL4, NAMPT, CXCR2, HBEGF* |
| GO:0042493 | response to drug | 6 | 0.014269 | *FOSL1, JUN, APOA2, FOSB, FOS, PTGS2* |
| GO:0007186 | G-protein coupled receptor signaling pathway | 10 | 0.024964 | *HCAR3, CXCL8, CXCR1, CCL20, CCL4, PROK2, FFAR2, CXCL1, CXCL3, CXCL2* |

**Supplementary table 7: The GO biological process analysis of blue module in SLE**

| Gene numbers | Gene terms | Counts | *P*-values | Genes |
| --- | --- | --- | --- | --- |
| GO:0008217 | regulation of blood pressure | 12 | 3.43E-05 | *CHGA, RAMP2, ACE, NPR1, NOS3, NPR3, NPY1R, UTS2, REN, PPARG, NOX1, CORIN* |
| GO:0006508 | proteolysis | 36 | 5.02E-04 | *FCN2, TPSB2, MYRF, PAPLN, ADAM22, KLK2, PHEX, ENDOU, PLAU, ADAMTS1, NRIP2, NAALAD2, OLR1, CTSG, PRSS2, ELANE, CPA5, MBL2, CELA2B, GGT6, CLCA3P, F11, PRSS36, AZU1, PRSS33, MMP8, TMPRSS11E, MMP9, PROC, LCN1, MMP19, TLL2, REN, PRTN3, KLKB1, LTF* |
| GO:0022617 | extracellular matrix disassembly | 11 | 6.48E-04 | *CDH1, CMA1, TLL2, MMP19, CTSG, KLK2, MMP8, KLKB1, PRSS2, MMP9, ELANE* |
| GO:0030574 | collagen catabolic process | 10 | 7.33E-04 | *COL3A1, ADAMTS3, COL5A1, MMP19, COL6A6, PRTN3, COL6A5, MMP8, PRSS2, MMP9* |
| GO:0007268 | chemical synaptic transmission | 21 | 0.001028 | *KCND2, GAD1, KCNC4, PCDH8, HTR1A, PMCHL2, PCDHB14, HTR4, CACNA1E, LRP6, HTR7, GALR2, NPBWR2, PMP22, KCNMB3, UTS2, APBA1, ASIC2, NPTX2, NTSR1, CBLN1* |
| GO:0006813 | potassium ion transport | 11 | 0.001181 | *KCNV1, KCNH4, KCNC4, KCNA2, ATP1A4, KCNMB3, ABCC9, KCNK1, SLC12A8, KCNJ2, KCNJ3* |
| GO:0042060 | wound healing | 10 | 0.003583 | *DSP, PDGFRA, COL3A1, TGFB2, WNT5B, ARHGEF19, EPPK1, GRHL3, FGF2, ITGA9* |
| GO:0034765 | regulation of ion transmembrane transport | 12 | 0.003685 | *KCNV1, KCNH4, KCND2, KCNJ9, KCNC4, KCNA2, CLCA3P, ASIC2, CACNA1E, NOX1, KCNJ3, SCN1A* |
| GO:0007399 | nervous system development | 22 | 0.003827 | *TIMM8A, CHRNA3, GSK3A, TRPC5, CHRM1, MYT1L, PCDHB15, INHBA, GFRA3, FGF2, TTLL7, OLFM1, DPYSL4, MDK, MPPED2, INSC, PCDHA5, APBA1, RAPGEF5, ZNF423, CBLN1, PCDHA6* |
| GO:0006952 | defense response | 9 | 0.004092 | *SPAG11B, SPAG11A, CRISP3, HP, INHBA, MPO, LILRA3, FOXN1, IRGM* |
| GO:0030317 | sperm motility | 8 | 0.004482 | *LDHC, SLC22A16, CATSPERD, NME8, CELF3, ATP1A4, TEKT2, PLTP* |
| GO:0050829 | defense response to Gram-negative bacterium | 8 | 0.004969 | *CHGA, FCN2, IL23R, DEFA4, RNASE7, BPI, AZU1, CAMP* |
| GO:0050830 | defense response to Gram-positive bacterium | 10 | 0.005373 | *CHGA, FCN2, DEFA4, RNASE7, C10ORF99, RNASE3, PGLYRP1, CAMP, EPHA2, MBL2* |
| GO:0008544 | epidermis development | 10 | 0.005373 | *DSP, COL17A1, BNC1, C1ORF68, GRHL3, KRT32, KRT5, KRT85, FOXN1, POU2F3* |
| GO:0001822 | kidney development | 10 | 0.005801 | *PYGO1, TGFB2, ACE, SALL1, WT1, ADAMTS1, SIX2, RGN, REN, C5ORF42* |
| GO:0016477 | cell migration | 15 | 0.00681 | *TGFB2, FOXE1, USP9Y, CDC42BPA, ARC, CEACAM1, COL5A1, MDK, SIX2, SNAI1, ERG, CD24, GPC6, NOX1, EPHA2* |
| GO:0030154 | cell differentiation | 30 | 0.006995 | *MYT1L, BNC1, ABCB5, SPIC, SLC22A16, MDK, ASZ1, INSC, AGR3, ZNF423, FGF23, MYBL1, PRM1, TMEM176B, PAX4, CEL, INHBA, NPAS4, TTLL7, SFRP5, SMOC1, CATSPERG, NME8, MGP, MMP19, TLL2, NANOG, ERG, TNP2, FOXA3* |
| GO:0045165 | cell fate commitment | 7 | 0.008068 | *NEUROD4, WNT5B, HEY2, SPRY2, PPARG, WNT16, WNT1* |
| GO:0008584 | male gonad development | 10 | 0.010249 | *TGFB2, KITLG, SIX4, WT1, TEX11, COL9A3, REN, NR0B1, INHBA, LHX9* |
| GO:0010628 | positive regulation of gene expression | 19 | 0.013053 | *TGFB2, CNTF, RAMP2, FOXD1, AVPR2, INHBA, AZU1, NGF, WNT16, ACTG2, DLL4, OLFM1, CYP26B1, CUX2, RPS6KA2, ID1, LCN2, SPRY2, STOX1* |
| GO:0045766 | positive regulation of angiogenesis | 11 | 0.013361 | *GREM1, RAMP2, ANXA3, NOS3, CMA1, ANGPTL3, CHI3L1, UTS2, FGF2, CAMP, RAPGEF3* |
| GO:0007218 | neuropeptide signaling pathway | 10 | 0.015911 | *UCN, GALR2, NPBWR2, NMU, NPY1R, GPR84, SORCS3, SORCS2, MCHR2, NTSR1* |
| GO:0001580 | detection of chemical stimulus involved in sensory perception of bitter taste | 6 | 0.018057 | *TAS2R40, TAS2R41, TAS2R13, TAS2R5, TAS2R19, TAS2R4* |
| GO:0048839 | inner ear development | 6 | 0.019932 | *TGFB2, BMPER, HPCA, PCDH15, STOX1, FREM2* |
| GO:0043408 | regulation of MAPK cascade | 6 | 0.019932 | *BMP3, ID1, REN, INHBA, CD24, INHBE* |
| GO:0007601 | visual perception | 15 | 0.023927 | *PDE6H, RGS16, PCDH15, TACSTD2, RORB, PDC, DLL4, RBP4, EFEMP1, GRK7, SFRP5, PDE6C, HMCN1, CRYAA, BBS5* |
| GO:0019731 | antibacterial humoral response | 6 | 0.026308 | *SLPI, DEFA4, RNASE7, RNASE3, CAMP, LTF* |
| GO:0007018 | microtubule-based movement | 8 | 0.036428 | *KIF19, KIF25, KIF24, KIF12, DYNLRB2, DNAH9, NGF, KIF27* |
| GO:0051384 | response to glucocorticoid | 7 | 0.038739 | *GHRHR, UCN, MDK, ANXA3, CDO1, S100B, AGXT* |
| GO:0007417 | central nervous system development | 10 | 0.042326 | *CHST8, AHI1, PPP1R17, SNTG2, ADAM22, NRCAM, GRHL3, ASIC2, S100B, ATOH1* |
| GO:0071805 | potassium ion transmembrane transport | 10 | 0.04424 | *KCNV1, KCNH4, KCND2, KCNC2, SLC9A7, KCNC4, KCNA2, KCNMB3, KCNK1, KCNJ2* |
| GO:0001764 | neuron migration | 9 | 0.04979 | *GPM6A, NRP1, NEUROD4, CHL1, DCC, NRCAM, GFRA3, KIAA0319, ATOH1* |

**Supplementary table 8: The GO biological process analysis of yellow module in SLE**

| GO numbers | GO terms | Counts | *P*-values | Genes |
| --- | --- | --- | --- | --- |
| GO:0008152 | metabolic process | 12 | 7.73E-04 | *PCSK1, TKTL1, AADAC, EPHX2, PDE2A, AFMID, UGT2B4, CPE, ACSM2A, ENPP6, ASPA, FTCDNL1* |
| GO:0007204 | positive regulation of cytosolic calcium ion concentration | 10 | 0.001918 | *EDNRB, GPER1, HRH4, C3AR1, CCR8, AVP, GATA2, TAC1, CCR4, CCR3* |
| GO:0021510 | spinal cord development | 5 | 0.003246 | *ROBO2, NOG, ZIC1, NEFL, NEUROG3* |
| GO:0007417 | central nervous system development | 9 | 0.003532 | *ROBO2, VNN1, CNTN6, ZBTB16, ID3, NES, TIMP4, NPAS2, NEUROG3* |
| GO:0007156 | homophilic cell adhesion via plasma membrane adhesion molecules | 10 | 0.005737 | *ROBO2, PCDHGB6, PCDHB2, CADM1, PCDHGA3, CADM2, FAT3, PCDHB3, PCDHA10, CDH7* |
| GO:0006954 | inflammatory response | 17 | 0.005755 | *CCL11, EPHX2, C3, TPST1, HRH1, VNN1, SCUBE1, GPER1, KIT, HRH4, C3AR1, TAC1, MS4A2, CCR4, TNFRSF4, TNFRSF21, CCR3* |
| GO:0007416 | synapse assembly | 6 | 0.008559 | *NLGN1, PCDHB2, BDNF, GJA10, PCDHB3, SPTBN2* |
| GO:0010628 | positive regulation of gene expression | 13 | 0.008719 | *PID1, EPHX2, NOG, FN1, CTCFL, RIMS2, AR, FGF9, GPER1, KIT, CNTN1, ITGB8, AVP* |
| GO:0008217 | regulation of blood pressure | 6 | 0.011117 | *POMC, EDNRB, ERAP2, NPY, EPHX2, TAC1* |
| GO:0007169 | transmembrane receptor protein tyrosine kinase signaling pathway | 7 | 0.014792 | *NTRK1, DOK4, FLT3, KIT, ROR1, CSPG4, FRK* |
| GO:0030178 | negative regulation of Wnt signaling pathway | 5 | 0.022497 | *DKK4, FGF9, FRZB, APCDD1, DKK3* |
| GO:0001558 | regulation of cell growth | 6 | 0.02523 | *IGFBP1, IGFBPL1, IGFBP2, HTRA4, HTRA1, WFDC1* |
| GO:0043410 | positive regulation of MAPK cascade | 6 | 0.026449 | *AR, FGF9, FLT3, GPER1, KIT, TNFRSF4* |
| GO:0007368 | determination of left/right symmetry | 5 | 0.027017 | *DYX1C1, DAAM2, DAW1, RPGRIP1L, PCSK6* |
| GO:0050680 | negative regulation of epithelial cell proliferation | 5 | 0.028636 | *CDKN1C, NKX2-8, AR, CDKN2B, WFDC1* |
| GO:0007155 | cell adhesion | 17 | 0.030425 | *ROBO2, SIGLEC16, CGREF1, NLGN1, CCL11, CNTN6, FN1, LYVE1, PCDHA10, IGSF11, PCDHB2, CNTN1, ITGB8, CCR8, PCDHB3, CNTN4, CCR3* |
| GO:0009612 | response to mechanical stimulus | 5 | 0.033832 | *IGFBP2, INHBB, KCNA5, SOST, KCNK2* |
| GO:0007267 | cell-cell signaling | 11 | 0.039467 | *POMC, NR5A1, PCSK1, AR, FGF9, BDNF, GDF15, ASIP, AVP, TAC1, AREG* |
| GO:0071805 | potassium ion transmembrane transport | 7 | 0.040187 | *KCNE1, KCNJ11, KCNS3, KCNK15, KCNA5, TMEM38A, KCNK2* |
| GO:0018108 | peptidyl-tyrosine phosphorylation | 8 | 0.040368 | *NTRK1, DYRK3, FGF9, FLT3, KIT, IL3RA, ROR1, MST1R* |
| GO:0009611 | response to wounding | 5 | 0.041559 | *FN1, ID3, SLC1A3, LYVE1, F2RL2* |
| GO:0006935 | chemotaxis | 7 | 0.041561 | *IL4, CCL11, C3AR1, CCR8, RNASE2, CCR4, CCR3* |
| GO:0043547 | positive regulation of GTPase activity | 19 | 0.047857 | *NTRK1, KLB, CCL11, RGS13, IQSEC3, ITSN1, ARHGAP29, RASAL1, FGF9, CDC42EP5, CHN1, KIT, IL3RA, NEFL, AMPH, SRGAP1, MCF2L2, SPTBN2, RAPGEF4* |

**Supplementary table 9: The GO biological process analysis of DEGs in SLE**

| Gene numbers | GO terms | Counts | *P*-values | Genes |
| --- | --- | --- | --- | --- |
| GO:0006955 | immune response | 38 | 1.27E-19 | *IFITM3, KIR3DS1, CCL4L2, NCF4, IFI6, FASLG, CTSW, KIR2DL1, CST7, KIR2DL3, VPREB3, IL18RAP, RGS1, CCL2, CTSG, DEFA1B, FCGR1A, PGLYRP1, FCGR1B, CD96, GZMA, KIR2DS5, KIR3DL1, DEFA1, GZMH, TGFBR3, TLR1, NCR3, OAS1, SLPI, OAS2, OAS3, CD7, BPI, CEACAM8, MS4A2, IL7R, IL18R1* |
| GO:0060337 | type I interferon signaling pathway | 16 | 6.61E-15 | *IFITM3, EGR1, RSAD2, MX2, MX1, IFI6, ISG15, IFI35, IFIT1, IFIT3, IFIT2, OASL, IFI27, OAS1, OAS2, OAS3* |
| GO:0051607 | defense response to virus | 21 | 1.23E-13 | *IFITM3, SPON2, RSAD2, MX2, MX1, PRF1, DEFA3, ISG15, DEFA1, AZU1, IFIT1, IFIT3, IFI44L, IFIT2, OASL, HERC5, PLSCR1, OAS1, OAS2, OAS3, DEFA1B* |
| GO:0045071 | negative regulation of viral genome replication | 11 | 1.18E-10 | *IFITM3, PLSCR1, RSAD2, OAS1, SLPI, OAS3, MX1, ISG15, IFIT1, LTF, OASL* |
| GO:0045087 | innate immune response | 27 | 2.57E-10 | *C1QB, SPON2, KIR3DS1, NCF2, HMGB2, HERC5, LGALS3, S100A12, DEFA1B, PGLYRP1, CAMP, KLRG1, ZNF683, KLRC2, DEFA4, MX2, MX1, KIR2DS5, SH2D1B, DEFA3, DEFA1, TLR1, SLPI, LCN2, ANG, KLRD1, PADI4* |
| GO:0050832 | defense response to fungus | 9 | 2.01E-09 | *SPON2, GNLY, DEFA4, DEFA3, S100A12, CTSG, DEFA1, DEFA1B, MPO* |
| GO:0050776 | regulation of immune response | 17 | 3.30E-09 | *COL17A1, CD96, KLRB1, CD160, SH2D1B, KIR3DL1, KIR3DL2, KIR2DL1, KIR2DL3, KIR2DL4, NCR3, CD19, KLRF1, KLRD1, KLRC1, CD247, FCGR1A* |
| GO:0019731 | antibacterial humoral response | 10 | 7.04E-09 | *SLPI, DEFA4, DEFA3, ADM, ANG, DEFA1, RNASE3, DEFA1B, CAMP, LTF* |
| GO:0009615 | response to virus | 13 | 3.70E-08 | *IFITM3, RSAD2, MX2, MX1, IFI44, IFIT1, IFIT3, IFIT2, OASL, OAS1, OAS2, OAS3, LCN2* |
| GO:0006968 | cellular defense response | 10 | 1.63E-07 | *LGALS3BP, KLRC2, NCF2, GNLY, CD160, CD19, PRF1, KIR3DL2, KIR2DL4, KLRG1* |
| GO:0031640 | killing of cells of other organism | 6 | 8.54E-07 | *GNLY, DEFA4, DEFA3, S100A12, DEFA1, DEFA1B* |
| GO:0050830 | defense response to Gram-positive bacterium | 10 | 2.51E-06 | *DEFA4, HMGB2, DEFA3, ADM, ANG, DEFA1, RNASE3, DEFA1B, PGLYRP1, CAMP* |
| GO:0002227 | innate immune response in mucosa | 6 | 2.00E-05 | *DEFA3, DEFA1, RNASE3, DEFA1B, CAMP, LTF* |
| GO:0001878 | response to yeast | 5 | 2.26E-05 | *ADM, ANG, MPO, CAMP, ELANE* |
| GO:0007166 | cell surface receptor signaling pathway | 15 | 2.74E-05 | *KLRB1, CD160, TACSTD2, ADRB2, CD2, IL18RAP, CD19, KLRF1, CCL2, KLRD1, KLRC1, CD247, IL7R, KLRG1, LY6E* |
| GO:0050829 | defense response to Gram-negative bacterium | 7 | 1.05E-04 | *CD160, DEFA4, HMGB2, ADM, BPI, AZU1, CAMP* |
| GO:0042742 | defense response to bacterium | 10 | 1.81E-04 | *TLR1, ANXA3, GNLY, HP, DEFA3, S100A12, ISG15, MPO, CAMP, ELANE* |
| GO:0019732 | antifungal humoral response | 4 | 2.87E-04 | *ADM, ANG, CAMP, LTF* |
| GO:0006954 | inflammatory response | 15 | 7.90E-04 | *ORM1, GPR68, CCL4L2, AZU1, FPR2, TLR1, NCR3, IL18RAP, C3AR1, S100A12, CCL2, OLR1, MS4A2, PTGDR, KLRG1* |
| GO:0044130 | negative regulation of growth of symbiont in host | 4 | 0.001261 | *CTSG, MPO, CAMP, ELANE* |
| GO:0050900 | leukocyte migration | 8 | 0.001495 | *CD2, CEACAM1, CEACAM6, C3AR1, OLR1, CEACAM8, FPR2, MMP9* |
| GO:0032496 | response to lipopolysaccharide | 9 | 0.001977 | *CD96, SLPI, HMGB2, FASLG, ADM, CTSG, TRIB1, MPO, ELANE* |
| GO:0060326 | cell chemotaxis | 6 | 0.001992 | *CCL4L2, HMGB2, C3AR1, CCL2, AZU1, FPR2* |
| GO:0019835 | cytolysis | 4 | 0.002846 | *GZMA, PRF1, GZMB, GZMH* |
| GO:0060333 | interferon-gamma-mediated signaling pathway | 6 | 0.002939 | *OAS1, OAS2, OAS3, FCGR1A, FCGR1B, OASL* |
| GO:0031100 | organ regeneration | 5 | 0.003916 | *TGFBR3, CCNA2, ANXA3, CCL2, ADM* |
| GO:0006508 | proteolysis | 16 | 0.004 | *C1QB, CPA3, GCA, AZU1, MMP8, PRSS23, MMP9, F5, MMP23B, OLR1, CTSG, PRTN3, LAP3, CTSD, ELANE, LTF* |
| GO:0071222 | cellular response to lipopolysaccharide | 7 | 0.004727 | *SPON2, ARG1, HMGB2, LCN2, CMPK2, CCL2, CAMP* |
| GO:0035457 | cellular response to interferon-alpha | 3 | 0.006365 | *OAS1, IFIT3, IFIT2* |
| GO:0070301 | cellular response to hydrogen peroxide | 5 | 0.007797 | *IL18RAP, PLEKHA1, ARG1, PDGFD, LCN2* |
| GO:0007204 | positive regulation of cytosolic calcium ion concentration | 7 | 0.010605 | *C3AR1, CD38, ADM, FPR2, GATA2, CD24, PTGDR* |
| GO:0048246 | macrophage chemotaxis | 3 | 0.0133 | *LGALS3, CCL2, AZU1* |
| GO:2000484 | positive regulation of interleukin-8 secretion | 3 | 0.0133 | *CD2, FFAR2, CAMP* |
| GO:0006952 | defense response | 5 | 0.013607 | *MX2, MX1, HP, TFF3, MPO* |
| GO:0071354 | cellular response to interleukin-6 | 3 | 0.015378 | *PID1, CCL2, CAMP* |
| GO:0006953 | acute-phase response | 4 | 0.016317 | *CD163, ORM1, PLSCR1, HP* |
| GO:0002250 | adaptive immune response | 7 | 0.016639 | *EOMES, ZNF683, TXK, CD7, SH2D1B, TNFRSF17, FCGR1B* |
| GO:0007165 | signal transduction | 26 | 0.017349 | *LGALS3BP, GRN, KIR3DS1, CLIC3, ADM, FASLG, RAP1GAP, KIR2DL4, RGS1, CCL2, TNFRSF17, CD38, FCGR1A, PRKCH, BCL11B, KLRC2, MX1, KIR2DS5, KIR3DL1, GNG11, TLR1, CEACAM6, IL2RB, ZNF831, IL7R, IL18R1* |
| GO:0007076 | mitotic chromosome condensation | 3 | 0.017584 | *CDCA5, NUSAP1, NCAPG* |
| GO:0002548 | monocyte chemotaxis | 4 | 0.019892 | *LGALS3, CCL4L2, S100A12, CCL2* |
| GO:0045454 | cell redox homeostasis | 5 | 0.02162 | *NCF2, NCF4, CAMP, LTF, TXNDC5* |
| GO:0008015 | blood circulation | 4 | 0.023864 | *C3AR1, OLR1, ADM, F5* |
| GO:0006334 | nucleosome assembly | 6 | 0.024354 | *H1F0, HMGB2, HIST1H4H, PADI4, HIST1H4E, HIST1H1C* |
| GO:0036120 | cellular response to platelet-derived growth factor stimulus | 3 | 0.024941 | *CCNA2, PDGFD, CCL2* |
| GO:0045089 | positive regulation of innate immune response | 3 | 0.024941 | *PLSCR1, SH2D1B, HMGB2* |
| GO:0006935 | chemotaxis | 6 | 0.026756 | *C3AR1, CCL2, DEFA1, FPR2, DEFA1B, RNASE2* |
| GO:0007568 | aging | 7 | 0.026779 | *CDKN1C, ARG1, CCL2, ADM, RETN, TYMS, MPO* |
| GO:0060700 | regulation of ribonuclease activity | 2 | 0.027325 | *OAS1, OAS3* |
| GO:0048285 | organelle fission | 2 | 0.027325 | *MX2, MX1* |
| GO:0002228 | natural killer cell mediated immunity | 2 | 0.027325 | *KLRC2, KLRD1* |
| GO:0050725 | positive regulation of interleukin-1 beta biosynthetic process | 2 | 0.027325 | *EGR1, AZU1* |
| GO:0030101 | natural killer cell activation | 3 | 0.030422 | *CD2, KIR3DS1, IL18R1* |
| GO:0045664 | regulation of neuron differentiation | 3 | 0.030422 | *EOMES, BCL11B, S1PR5* |
| GO:0016477 | cell migration | 7 | 0.031866 | *TGFBR3, CEACAM1, BAMBI, PALLD, ANG, CD24, PARP9* |
| GO:0001666 | response to hypoxia | 7 | 0.031866 | *TGFBR3, EGR1, CD38, CCL2, ADM, ANG, CD24* |
| GO:0006401 | RNA catabolic process | 3 | 0.033325 | *OAS2, RNASE3, RNASE2* |
| GO:0030520 | intracellular estrogen receptor signaling pathway | 3 | 0.033325 | *DEFA3, DEFA1, DEFA1B* |
| GO:0045648 | positive regulation of erythrocyte differentiation | 3 | 0.042641 | *HMGB2, ISG15, GATA2* |
| GO:0006898 | receptor-mediated endocytosis | 7 | 0.043866 | *LGALS3BP, CD163, HP, OLR1, ADRB2, FCGR1A, FCGR1B* |
| GO:0048589 | developmental growth | 3 | 0.045938 | *ASPM, ADM, TYMS* |
| GO:0060135 | maternal process involved in female pregnancy | 3 | 0.045938 | *IFI27, ARG1, CCL2* |
| GO:0043406 | positive regulation of MAP kinase activity | 4 | 0.047597 | *PDGFD, S100A12, CD24, ELANE* |

**Supplementary table 10: The GO biological process analysis of DEGs in PAH**

| Gene numbers | Gene terms | Counts | *P*-Value | | Genes |
| --- | --- | --- | --- | --- | --- |
| GO:0006954 | inflammatory response | 38 | 2.31E-10 | *ECM1, PTGER2, C5AR1, PTAFR, CXCL1, FPR2, C4B_2, PTGS2, TNF, CXCL5, C4B, NDST1, C4A, C5, CASP4, STAB1, C3AR1, S100A12, CCR7, CD14, CCR1, CCL25, GGT5, IL10RB, IL15, NMI, TNFRSF1B, HCK, CD40LG, TNIP1, BCL6, IL1B, ADAM8, PTX3, LTBR, S100A9, TLR4, MYD88* | |
| GO:0007165 | signal transduction | 73 | 2.05E-09 | *GABRB1, ECM1, PDE3B, ARHGAP1, SECTM1, ADM, CBLB, CXCL1, SHB, ADRA1A, SIRPB1, CXCL5, NR3C2, THBD, FGF6, PPP4R1, LGALS1, RGS1, DPYSL2, TNFSF10, CD38, OSTF1, PRKACB, MAP2K3, DFFA, IFNGR1, IL15, PLAUR, CREB1, MAPKAPK3, IL1B, RASA2, SAG, S100A9, MAPRE2, KHDRBS1, PRKAA1, GRN, SDC4, CHRNA4, EPAS1, C5AR1, ARHGAP19, PIK3R1, TXN, LILRA2, TANK, STK3, ABR, GNG10, MTA1, IRAK1, IL12RB1, FCGR1A, MPP1, IL10RB, VDR, MX1, LILRB1, SORL1, EXT1, MAPK11, P2RX4, RPS6KB1, RHEB, TRIP10, ZYX, PKN2, ATM, LTBR, PDE7A, IL7R, MYD88* | |
| GO:0030449 | regulation of complement activation | 11 | 8.81E-09 | *C4B, C4A, C5, C5AR1, C3AR1, CD59, CD46, C4B_2, CFP, CD55, C2* | |
| GO:0060333 | interferon-gamma-mediated signaling pathway | 15 | 1.82E-08 | *IFNGR1, STAT1, IFNGR2, PTAFR, NMI, IFI30, ICAM1, HCK, MT2A, IRF1, IRF7, IRF5, FCGR1A, FCGR1B, GBP1* | |
| GO:0031663 | lipopolysaccharide-mediated signaling pathway | 10 | 2.61E-07 | *NFKBIA, HCK, IRAK1, IL1B, PTAFR, CD14, TNF, TLR4, MYD88, MAPK3* | |
| GO:0043066 | negative regulation of apoptotic process | 35 | 8.81E-07 | *PRKAA1, BCL2A1, GATA6, HTR2B, CIB1, PIK3R1, YBX3, IFIT3, BAG6, PRDX3, IRAK1, UBB, BAG1, CD38, CD59, PIM2, NTSR1, TFAP2B, PRNP, PRKCI, PLAUR, PAFAH2, BTC, NFKBIA, HCK, CD40LG, RPS6KB1, BCL3, FKBP8, PPT1, MDM4, IL6ST, SQSTM1, MYD88, PPARD* | |
| GO:0009615 | response to virus | 16 | 9.10E-07 | *MEF2C, IFNGR1, IFNGR2, MX1, IFI44, DDX21, LILRB1, IFIT1, TNF, IFIT3, FGR, FOSL1, BCL3, IRF7, PIM2, MYD88* | |
| GO:0045087 | innate immune response | 33 | 2.02E-06 | *NCF2, CD1D, C4B_2, SIRPB1, C2, C4B, C4A, LGALS3, IRAK1, UBB, CASP4, S100A12, CSK, CD14, IGHA1, IGHA2, CD55, MAP3K5, MAP4K2, TNK1, MX1, S100B, FGR, IRF7, BTK, SERPING1, PTX3, SERINC3, CD46, S100A9, TLR4, MYD88, APOBEC3B* | |
| GO:0006955 | immune response | 32 | 3.64E-06 | *LST1, NCF4, C5AR1, PTAFR, SECTM1, CXCL1, CFP, ETS1, TNF, CXCL5, NFIL3, RGS1, FTH1, TNFSF10, CCR7, FCGR1A, IGHA1, FCGR1B, IGHA2, CCR1, CCL25, MAP4K2, IL10RB, IL15, OPRK1, TNFRSF1B, CHIT1, CD40LG, IL1B, LTBR, IL7R, TLR4* | |
| GO:0032496 | response to lipopolysaccharide | 18 | 8.20E-06 | *JUND, PTGER2, C5AR1, ADM, CXCL1, PTGS2, CXCL5, PRDX3, THBD, ABR, MTA1, IRAK1, RPS6KB1, MAPKAPK3, CCR7, LTBR, TLR4, TIMP4* | |
| GO:0051092 | positive regulation of NF-kappaB transcription factor activity | 16 | 1.00E-05 | *PRKCI, CIB1, TNF, ICAM1, PRDX3, NFKBIA, CD40LG, IRAK1, UBB, IL1B, BTK, S100A12, ADAM8, S100A9, TLR4, MYD88* | |
| GO:0010575 | positive regulation of vascular endothelial growth factor production | 8 | 1.05E-05 | *C5, IL1B, C5AR1, C3AR1, CYP1B1, IL6ST, PTGS2, ISL1* | |
| GO:0050727 | regulation of inflammatory response | 11 | 1.55E-05 | *HCK, MTA1, BCL6, TNIP1, CASP4, AGTR1, ZYX, NR1D2, PTGS2, MYD88, SLC7A2* | |
| GO:0000187 | activation of MAPK activity | 14 | 1.75E-05 | *MAP2K3, PRKAA1, HGF, C5AR1, TNF, MAPK11, C5, IRAK1, UBB, MAPKAPK3, IL1B, TLR4, DRD4, MAPK3* | |
| GO:0045893 | positive regulation of transcription, DNA-templated | 34 | 3.19E-05 | *RNF10, SMARCD3, CHD8, CD80, GATA6, AHR, ETS1, TNF, NPAT, IRAK1, HNF4A, CD38, ALX1, PIM2, MYBL1, MAPK3, NCOA1, MAP2K3, NFE2, TFAP2B, ZFHX3, MEF2C, BRPF1, STAT1, RHOG, NR1D2, KLF6, CREB1, IRF1, IL1B, BCL3, IRF7, MAPRE3, PPARD* | |
| GO:0043065 | positive regulation of apoptotic process | 24 | 3.51E-05 | *NCOA1, DFFA, BCL2A1, MCF2, ADM, LILRB1, S100B, PTGS2, TNF, STK3, FOSL1, ABR, CREB1, UBB, BCL6, TNFSF10, CYP1B1, HMOX1, MNDA, IRF5, ATM, SQSTM1, NTSR1, MAP3K5* | |
| GO:0008285 | negative regulation of cell proliferation | 28 | 5.94E-05 | *TSG101, CTBP1, ADM, CIB1, CXCL1, TOB2, PTGS2, ETS1, ADRA1A, IFIT3, STK3, HNF4A, FTH1, MXI1, TIMP2, CYP1B1, CSK, PIM2, KLF10, TFAP2B, VDR, SSTR1, FOSL1, BCL6, IRF1, IL1B, MDM4, NF2* | |
| GO:0043123 | positive regulation of I-kappaB kinase/NF-kappaB signaling | 16 | 9.54E-05 | *ECM1, HTR2B, SECTM1, S100B, TNF, LGALS1, IRAK1, UBB, TNFSF10, S100A13, S100A12, HMOX1, CCR7, PIM2, LTBR, MYD88* | |
| GO:0060337 | type I interferon signaling pathway | 10 | 1.10E-04 | *IFI27, STAT1, IRF1, MX1, IRF7, ISG15, IRF5, IFI35, IFIT1, IFIT3* | |
| GO:0042493 | response to drug | 23 | 1.21E-04 | *APOBEC1, TFAP2B, JUND, SLC12A5, ACSL1, STAT1, SRD5A1, ABCA3, GATA6, HTR2B, AK4, PTGS2, ADRA1A, ICAM1, FOSL1, CREB1, LGALS1, SRP72, RPS6KB1, DPYSL2, TIMP2, CD38, TIMP4* | |
| GO:0046330 | positive regulation of JNK cascade | 10 | 1.24E-04 | *SDCBP, MAP4K2, IL1B, CCR7, LTBR, TNF, TLR4, MYD88, STK3, MAP3K5* | |
| GO:0060326 | cell chemotaxis | 10 | 1.24E-04 | *CCL25, C5, HGF, C5AR1, RHOG, AGTR1, C3AR1, CXCL1, FPR2, CXCL5* | |
| GO:0071456 | cellular response to hypoxia | 12 | 1.31E-04 | *PRKAA1, IRAK1, EPAS1, GATA6, HMOX1, MDM4, ADAM8, S100B, PTGS2, ICAM1, BBC3, PPARD* | |
| GO:0042127 | regulation of cell proliferation | 17 | 1.36E-04 | *APOBEC1, JUND, PTGER2, TNK1, CIB1, TNFRSF1B, TNF, SAT1, BAG6, NFKBIA, FGR, BCL6, BTK, AGTR1, CSK, LTBR, EZH2* | |
| GO:0007204 | positive regulation of cytosolic calcium ion concentration | 14 | 1.84E-04 | *CCR1, PTGER2, C5AR1, OPRL1, ADM, FPR2, ADRA1A, GNA15, CCKAR, C3AR1, AGTR1, CD38, CCR7, CD55* | |
| GO:0006915 | apoptotic process | 34 | 1.99E-04 | *C5AR1, CIB1, AHR, SHB, ADRA1A, STK3, BBC3, PRDX3, CASP7, LGALS1, CASP4, BAG1, TNFSF10, EP300, PIM2, CD14, MAPK3, TFAP2B, DFFA, MEF2C, STAT1, MX1, MFSD10, NFKBIA, RPS6KB1, IRF1, IL1B, FKBP8, PKN2, LTBR, S100A9, SQSTM1, MYD88, PPARD* | |
| GO:0000122 | negative regulation of transcription from RNA polymerase II promoter | 40 | 2.39E-04 | *ZNF177, ATF2, NOTCH3, CTBP1, CHD8, GATA6, AEBP1, TXN, YBX3, TNF, ZFP36, UBB, NFIL3, CUX1, ZNF280B, EP300, ALX1, HIST1H1C, KLF10, LMO1, HBZ, TFAP2B, ZFHX3, MEF2C, JUND, STAT1, VDR, S100A1, OLIG2, ISL1, ZNF559-ZNF177, BCL6, IFI27, IRF7, MDM4, ZNF136, SQSTM1, ATF3, PPARD, EZH2* | |
| GO:0097190 | apoptotic signaling pathway | 10 | 2.47E-04 | *P2RX4, IFI27, BTK, CD38, CD14, TNFRSF1B, TLR4, MAP3K5, BBC3, PPARD* | |
| GO:0006935 | chemotaxis | 13 | 2.84E-04 | *CCL25, CCR1, C5AR1, PTAFR, PLAUR, CXCL1, FPR2, CXCL5, FOSL1, C5, C3AR1, ACKR3, CCR7* | |
| GO:0050900 | leukocyte migration | 13 | 2.84E-04 | *ITGAM, ITGB3, C5AR1, ATP1B3, PIK3R1, FPR2, ICAM1, SLC7A5, THBD, ITGAX, C3AR1, SIRPA, CD58* | |
| GO:0007155 | cell adhesion | 29 | 2.88E-04 | *CD151, ITGAM, PTPRS, ITGB3, ATP2A2, CIB1, FPR2, HAPLN1, ICAM1, CDH4, STAB1, CTNNA1, ITGAX, CYP1B1, SIRPA, CD58, CCR1, EPHA4, OMD, GP5, HCK, VCAN, NINJ1, ITGA8, ZYX, ACKR3, ADAM9, CDH15, VCL* | |
| GO:0006366 | transcription from RNA polymerase II promoter | 31 | 3.59E-04 | *ATF2, EPAS1, ONECUT1, GATA6, FOXK2, DDX21, AHR, ETS1, NFIL3, EP300, TCEB1, ALX1, MYBL1, SOX5, TFAP2B, ZFHX3, MEF2C, JUND, FOXD1, VEZF1, NMI, ISL1, ETV5, FOSL1, MEIS1, CREB1, IRF1, IRF7, ZNF136, ATF3, TAF1* | |
| GO:0048661 | positive regulation of smooth muscle cell proliferation | 9 | 3.83E-04 | *NOTCH3, RPS6KB1, IRAK1, STAT1, PTAFR, HMOX1, PTGS2, TNF, MYD88* | |
| GO:0051607 | defense response to virus | 15 | 4.27E-04 | *APOBEC1, IL10RB, STAT1, MX1, OPRK1, ISG15, LILRB1, IFIT1, IFIT3, IRF1, ITGAX, IRF5, SERINC3, GBP1, APOBEC3B* | |
| GO:0045944 | positive regulation of transcription from RNA polymerase II promoter | 49 | 4.95E-04 | *RNF10, ATF2, HEXB, CHD8, ONECUT1, AHR, ETS1, TNF, HNF4A, EP300, MYBL1, LMO1, NCOA1, MEF2C, FOXD1, HGF, LMO2, ISL1, ETV5, CREB1, IRF1, IL1B, IRF7, IRF5, SQSTM1, TLR4, ATF3, NOTCH3, EPAS1, GATA6, FOXK2, PIK3R1, PPP3R1, UBB, ALX1, MAPK3, TFAP2B, JUND, STAT1, VDR, LILRB1, NFKBIA, FOSL1, MEIS1, MC1R, TNIP1, BCL3, EZH1, TAF1* | |
| GO:0071222 | cellular response to lipopolysaccharide | 12 | 5.51E-04 | *MEF2C, ZFP36, CD80, ADAM9, OPRK1, LILRB1, CD14, TNFRSF1B, TNF, TLR4, ICAM1, PPARD* | |
| GO:0007166 | cell surface receptor signaling pathway | 20 | 5.87E-04 | *CCL25, CCR1, KHDRBS1, CD151, NPY6R, IFNGR2, CBLB, SSTR1, SIRPB1, ASGR2, C5, MAPKAPK3, BAG1, TNFSF10, SAG, CD59, CD14, IL7R, MYD88, CD101* | |
| GO:0045766 | positive regulation of angiogenesis | 12 | 6.39E-04 | *ECM1, C5, IL1B, HGF, C5AR1, GATA6, C3AR1, CYP1B1, HMOX1, ADM, ETS1, ISL1* | |
| GO:0007200 | phospholipase C-activating G-protein coupled receptor signaling pathway | 9 | 7.37E-04 | *CCKAR, P2RY2, C5AR1, AGTR1, HTR2B, C3AR1, OPRK1, FPR2, ADRA1A* | |
| GO:0034097 | response to cytokine | 8 | 8.34E-04 | *FOSL1, JUND, IFI27, MAPKAPK3, STAT1, TIMP2, IL6ST, TIMP4* | |
| GO:0006468 | protein phosphorylation | 27 | 0.001252 | *PRKAA1, CTBP1, PIK3R1, STK3, PPP4R1, IRAK1, CSK, PIM2, ST3GAL1, PRKACB, MAPK3, MAP3K5, MAP4K2, PRKCI, DMPK, LIMK2, TNK1, FGR, HCK, CREB1, RPS6KB1, BTK, PKN2, ATM, SQSTM1, TAF1, MAP3K12* | |
| GO:0016032 | viral process | 20 | 0.001645 | *YWHAE, EIF4A1, YWHAB, PIK3R1, CD1D, PSMB9, NFKBIA, HCK, CREB1, ZYX, FKBP8, ACKR3, EP300, TCEB1, LTBR, CDK13, IL6ST, TAF1, MAP3K5, MAPK3* | |
| GO:0043406 | positive regulation of MAP kinase activity | 8 | 0.00178 | *GH1, MEF2C, IRAK1, HTR2B, S100A12, CSK, TNF, EZH2* | |
| GO:0000165 | MAPK cascade | 18 | 0.00228 | *MAP2K3, MEF2C, YWHAB, TNF, SPTB, PSMB9, BTC, MAPK11, FGF6, NDST1, ZFP36, UBB, MAPKAPK3, IL1B, RASA2, PSME2, MAP3K5, MAPK3* | |
| GO:0006958 | complement activation, classical pathway | 10 | 0.002765 | *C4B, C4A, C5, SERPING1, CD46, C4B_2, IGHA1, IGHA2, CD55, C2* | |
| GO:0001525 | angiogenesis | 16 | 0.002886 | *ECM1, EPAS1, PDE3B, TNFAIP2, VEZF1, CIB1, PTGS2, SHB, SAT1, FGF6, MEIS1, KRIT1, CYP1B1, ACKR3, HMOX1, ADAM8* | |
| GO:0008284 | positive regulation of cell proliferation | 26 | 0.003451 | *PRKAA1, HTR2B, ADM, CIB1, TCIRG1, ETS1, CXCL5, PRDX3, FGF6, SDCBP, HLX, S100A13, TFAP2B, IL15, HGF, RHOG, UBE2A, S100B, ISL1, BTC, FOSL1, HCK, CDK13, IL6ST, ATF3, TAF1* | |
| GO:0007596 | blood coagulation | 14 | 0.003515 | *NFE2, ITGB3, CYP4F2, GATA6, PLAUR, GP5, SERPINA5, THBD, P2RX4, HNF4A, IRF1, CD59, VPS45, PRKACB* | |
| GO:0007568 | aging | 13 | 0.003926 | *JUND, BCL2A1, IL15, ADM, TNFRSF1B, ADRA1A, CASP7, CREB1, IRAK1, RPS6KB1, TIMP2, CTNNA1, SERPING1* | |
| GO:0035556 | intracellular signal transduction | 23 | 0.004824 | *YWHAE, MAP4K2, DGKE, PRKCI, PRKAA1, S100A1, MCF2, CD80, CXCL1, ADRA1A, STK3, MAPK11, SDCBP, ABR, ZFP36, MC1R, RASA2, BTK, FKBP8, HMOX1, PKN2, SQSTM1, MAP3K12* | |
| GO:0030036 | actin cytoskeleton organization | 11 | 0.005523 | *SDCBP, ABR, BCL6, FLII, ARPC1A, FSCN1, RHOG, TRIP10, CXCL1, NF2, PDLIM7* | |
| GO:0048010 | vascular endothelial growth factor receptor signaling pathway | 8 | 0.005525 | *MAPK11, CYFIP1, MAPKAPK3, NCF2, ITGB3, NCF4, PIK3R1, SHB* | |
| GO:0007268 | chemical synaptic transmission | 16 | 0.00567 | *PTPRS, SLC12A5, CHRNA4, OPRL1, HTR2B, MYO5A, GPR1, OPRK1, SSTR1, SYN2, PTPRD, SDCBP, NOVA1, PPT1, SNCB, NTSR1* | |
| GO:0050729 | positive regulation of inflammatory response | 8 | 0.005959 | *TNIP1, IL15, AGTR1, S100A12, ETS1, S100A9, TLR4, LDLR* | |
| GO:0070374 | positive regulation of ERK1 and ERK2 cascade | 13 | 0.006251 | *CCL25, CCR1, ATP6AP1, C5AR1, HTR2B, CIB1, ADRA1A, TNF, ICAM1, ACKR3, CCR7, TLR4, MAPK3* | |
| GO:0050821 | protein stabilization | 11 | 0.007535 | *BAG6, CREB1, LAMP2, EP300, MDM4, ATP1B3, PIM2, PIK3R1, IFI30, STK3, TAF1* | |
| GO:0007229 | integrin-mediated signaling pathway | 9 | 0.009398 | *FGR, HCK, ITGAM, ITGB3, ITGA8, ITGAX, ZYX, ADAM9, ADAM8* | |
| GO:1901796 | regulation of signal transduction by p53 class mediator | 10 | 0.011867 | *MAPK11, PRKAA1, BRPF1, UBB, RPA1, EP300, MDM4, ATM, TAF1, RAD9A* | |
| GO:0046777 | protein autophosphorylation | 12 | 0.014089 | *FGR, EPHA4, HCK, SMG1, IRAK1, MAPKAPK3, TNK1, CSK, ATM, PIM2, TAF1, MAP3K12* | |
| GO:0001666 | response to hypoxia | 12 | 0.014089 | *ALAS2, PRKAA1, CREB1, CHRNA4, EPAS1, SLC11A2, EP300, CD38, HMOX1, ADM, ATM, ETS1* | |
| GO:0032355 | response to estradiol | 8 | 0.018822 | *NCOA1, GH1, IFI27, OPRL1, CD38, PTGS2, ETS1, EZH2* | |
| GO:0007267 | cell-cell signaling | 15 | 0.020339 | *CCR1, KLF10, IL15, CD80, ADM, TXN, SSTR1, ADRA1A, CXCL5, FGF6, IL1B, STAB1, TNFSF10, ZYX, S100A9* | |
| GO:0006874 | cellular calcium ion homeostasis | 8 | 0.020955 | *CCR1, DMPK, VDR, HEXB, HTR2B, ATP2B4, ATP2A2, DRD4* | |
| GO:0008584 | male gonad development | 8 | 0.022083 | *NCOA1, HOXA9, PRDX4, SRD5A1, GATA6, CTNNA1, TNFSF10, YBX3* | |
| GO:0006974 | cellular response to DNA damage stimulus | 13 | 0.022229 | *ATF2, USP10, CIB1, TANK, BBC3, BCL6, BCL3, IRF7, MNDA, ATM, TAF1, MAPK3, RAD9A* | |
| GO:0006898 | receptor-mediated endocytosis | 12 | 0.023716 | *STAB1, PPT1, CD14, FCGR1A, IGHA1, FCGR1B, IGHA2, SORL1, LDLR, ASGR1, PDLIM7, ASGR2* | |
| GO:0008360 | regulation of cell shape | 10 | 0.024288 | *FGR, CYFIP1, HCK, EPB42, HEXB, TPM1, LST1, S100A13, S100B, ICAM1* | |
| GO:0010628 | positive regulation of gene expression | 15 | 0.025739 | *MEF2C, PRKAA1, GSN, FOXD1, VDR, LILRB1, TNF, MAPK11, NFIL3, IL1B, CD46, IL7R, LDLR, TLR4, PPARD* | |
| GO:0010951 | negative regulation of endopeptidase activity | 9 | 0.02825 | *C4B, C4A, C5, SERPINB1, TIMP2, SERPING1, SERPINA6, TIMP4, SERPINA5* | |
| GO:0016477 | cell migration | 11 | 0.033373 | *FGR, CD63, PRKCI, CD151, SDC4, RPS6KB1, ITGB3, FSCN1, ADAM9, CSK, AVL9* | |
| GO:0018105 | peptidyl-serine phosphorylation | 9 | 0.033385 | *PRKCI, SMG1, DMPK, MAPKAPK3, PKN2, ATM, TAF1, MAP3K12, MAPK3* | |
| GO:0006357 | regulation of transcription from RNA polymerase II promoter | 21 | 0.041453 | *ATF2, NFE2, ECM1, SMARCD3, JUND, PTAFR, ATP2B4, IGHMBP2, FOXK2, CDC5L, VEZF1, AHR, ISL1, FOSL1, CREB1, CUX1, HNF4A, TCEB1, IRF5, ATF3, PPARD* | |
| GO:0043524 | negative regulation of neuron apoptotic process | 9 | 0.04383 | *TFAP2B, MEF2C, PRKCI, C5AR1, PPT1, HMOX1, ADAM8, SNCB, ISL1* | |
| GO:0045892 | negative regulation of transcription, DNA-templated | 23 | 0.043963 | *KLF10, KHDRBS1, TFAP2B, ZFHX3, TSG101, FOXD1, YWHAB, CTBP1, VDR, CHD8, GATA6, NR1D2, AHR, TNF, BCL6, IRF1, ZNF280B, BCL3, CD38, ALX1, EZH2, PPARD, RUNX1T1* | |
| GO:0006629 | lipid metabolic process | 10 | 0.045478 | *APOBEC1, ASAH1, CLPS, PITPNB, HNF4A, ALDH3B1, HSD17B10, LDLR, PAFAH2, PPARD* | |
| GO:0071356 | cellular response to tumor necrosis factor | 8 | 0.046127 | *CCL25, ZFP36, CIB1, CD58, YBX3, TANK, ICAM1, MAP3K5* | |

**Supplementary table 11: The GO biological process analysis of common miRNAs in PAH and SLE**

| GO Category | *P*-values | | Genes | miRNAs |
| --- | --- | --- | --- | --- |
| protein complex assembly | 0 | 183 | | 3 |
| transcription, DNA-templated | 0 | 548 | | 4 |
| blood coagulation | 0 | 148 | | 4 |
| cell death | 0 | 284 | | 4 |
| cellular component assembly | 0 | 342 | | 4 |
| nucleobase-containing compound catabolic process | 0 | 271 | | 4 |
| cellular protein metabolic process | 0 | 160 | | 4 |
| small molecule metabolic process | 0 | 613 | | 4 |
| macromolecular complex assembly | 0 | 250 | | 4 |
| DNA metabolic process | 0 | 241 | | 5 |
| catabolic process | 0 | 564 | | 5 |
| neurotrophin TRK receptor signaling pathway | 0 | 97 | | 5 |
| membrane organization | 0 | 193 | | 5 |
| mitotic cell cycle | 0 | 172 | | 6 |
| immune system process | 0 | 417 | | 6 |
| biological_process | 0 | 3902 | | 6 |
| biosynthetic process | 0 | 1213 | | 6 |
| gene expression | 0 | 283 | | 6 |
| cellular nitrogen compound metabolic process | 0 | 1493 | | 6 |
| cellular protein modification process | 0 | 787 | | 7 |
| response to stress | 0 | 672 | | 7 |
| viral process | 0 | 208 | | 7 |
| Fc-epsilon receptor signaling pathway | 0 | 65 | | 7 |
| symbiosis, encompassing mutualism through parasitism | 0 | 228 | | 7 |
| epidermal growth factor receptor signaling pathway | 6.77E-15 | 65 | | 4 |
| transcription initiation from RNA polymerase II promoter | 7.77E-15 | 74 | | 4 |
| G2/M transition of mitotic cell cycle | 1.12E-14 | 63 | | 4 |
| innate immune response | 9.40E-14 | 193 | | 5 |
| post-translational protein modification | 1.38E-13 | 62 | | 4 |
| mRNA metabolic process | 2.06E-12 | 73 | | 4 |
| cellular lipid metabolic process | 2.96E-11 | 47 | | 3 |
| Fc-gamma receptor signaling pathway involved in phagocytosis | 9.27E-11 | 33 | | 4 |
| cell cycle | 1.98E-10 | 218 | | 3 |
| activation of signaling protein activity involved in unfolded protein response | 7.07E-10 | 24 | | 3 |
| toll-like receptor TLR1:TLR2 signaling pathway | 7.31E-10 | 27 | | 3 |
| toll-like receptor TLR6:TLR2 signaling pathway | 7.31E-10 | 27 | | 3 |
| cytoskeleton organization | 9.68E-10 | 152 | | 3 |
| cellular component movement | 1.79E-09 | 33 | | 2 |
| toll-like receptor 9 signaling pathway | 3.29E-09 | 32 | | 4 |
| mRNA processing | 3.88E-09 | 149 | | 4 |
| toll-like receptor 10 signaling pathway | 4.50E-09 | 25 | | 3 |
| toll-like receptor signaling pathway | 5.93E-09 | 43 | | 4 |
| cellular component disassembly involved in execution phase of apoptosis | 6.52E-09 | 19 | | 2 |
| TRIF-dependent toll-like receptor signaling pathway | 6.78E-09 | 31 | | 4 |
| fibroblast growth factor receptor signaling pathway | 1.55E-08 | 48 | | 3 |
| platelet activation | 2.17E-08 | 57 | | 3 |
| vesicle-mediated transport | 2.18E-08 | 146 | | 2 |
| RNA metabolic process | 5.85E-08 | 64 | | 3 |
| RNA splicing | 6.98E-08 | 100 | | 4 |
| transforming growth factor beta receptor signaling pathway | 8.51E-08 | 55 | | 3 |
| toll-like receptor 5 signaling pathway | 1.23E-07 | 25 | | 3 |
| positive regulation of protein insertion into mitochondrial membrane involved in apoptotic signaling pathway | 1.33E-07 | 16 | | 2 |
| MyD88-independent toll-like receptor signaling pathway | 1.72E-07 | 31 | | 4 |
| insulin receptor signaling pathway | 2.05E-07 | 47 | | 3 |
| toll-like receptor 4 signaling pathway | 2.06E-07 | 23 | | 2 |
| intrinsic apoptotic signaling pathway | 2.45E-07 | 26 | | 2 |
| toll-like receptor 2 signaling pathway | 7.34E-07 | 28 | | 3 |
| chromatin organization | 7.96E-07 | 35 | | 3 |
| G1/S transition of mitotic cell cycle | 9.58E-07 | 53 | | 3 |
| nucleocytoplasmic transport | 1.04E-06 | 78 | | 2 |
| nucleotide-binding domain, leucine rich repeat containing receptor signaling pathway | 1.33E-06 | 15 | | 2 |
| phosphatidylinositol-mediated signaling | 1.41E-06 | 30 | | 2 |
| type I interferon signaling pathway | 1.92E-06 | 22 | | 2 |
| toll-like receptor 3 signaling pathway | 2.45E-06 | 28 | | 3 |
| cell cycle arrest | 4.72E-06 | 53 | | 3 |
| protein N-linked glycosylation via asparagine | 6.24E-06 | 30 | | 2 |
| leukocyte migration | 7.52E-06 | 34 | | 3 |
| transcription from RNA polymerase II promoter | 9.53E-06 | 111 | | 2 |
| cytokine-mediated signaling pathway | 1.06E-05 | 20 | | 1 |
| stress-activated MAPK cascade | 1.18E-05 | 18 | | 2 |
| regulation of transcription from RNA polymerase II promoter in response to hypoxia | 2.09E-05 | 10 | | 2 |
| platelet degranulation | 5.37E-05 | 14 | | 1 |
| phospholipid metabolic process | 5.58E-05 | 35 | | 2 |
| extracellular matrix disassembly | 6.14E-05 | 26 | | 2 |
| termination of RNA polymerase II transcription | 8.68E-05 | 18 | | 2 |
| negative regulation of transcription from RNA polymerase II promoter | 8.90E-05 | 123 | | 1 |
| post-Golgi vesicle-mediated transport | 0.000112555 | 11 | | 1 |
| nuclear-transcribed mRNA catabolic process, deadenylation-dependent decay | 0.000117675 | 16 | | 2 |
| cell junction organization | 0.00017234 | 35 | | 2 |
| axon guidance | 0.000281583 | 75 | | 2 |
| negative regulation of apoptotic process | 0.000383039 | 66 | | 1 |
| apoptotic signaling pathway | 0.000407418 | 35 | | 2 |
| endoplasmic reticulum unfolded protein response | 0.000712662 | 23 | | 1 |
| MyD88-dependent toll-like receptor signaling pathway | 0.000784703 | 20 | | 2 |
| protein ubiquitination | 0.00079038 | 76 | | 1 |
| mRNA 3'-end processing | 0.001386136 | 10 | | 1 |
| platelet-derived growth factor receptor signaling pathway | 0.001516583 | 19 | | 2 |
| cell proliferation | 0.001595736 | 73 | | 1 |
| mRNA splicing, via spliceosome | 0.002081335 | 14 | | 1 |
| cell motility | 0.002398974 | 54 | | 1 |
| DNA damage response, signal transduction by p53 class mediator resulting in cell cycle arrest | 0.003111979 | 11 | | 1 |
| JAK-STAT cascade involved in growth hormone signaling pathway | 0.003122151 | 10 | | 2 |
| protein polyubiquitination | 0.003173531 | 26 | | 1 |
| in utero embryonic development | 0.003527013 | 49 | | 1 |
| nucleotide-binding oligomerization domain containing signaling pathway | 0.003997929 | 6 | | 1 |
| glycerophospholipid biosynthetic process | 0.005667993 | 13 | | 1 |
| regulation of ubiquitin-protein ligase activity involved in mitotic cell cycle | 0.00588915 | 11 | | 1 |
| nuclear-transcribed mRNA poly(A) tail shortening | 0.006453441 | 10 | | 1 |
| positive regulation of ubiquitin-protein ligase activity involved in mitotic cell cycle | 0.007523811 | 11 | | 1 |
| negative regulation of cell proliferation | 0.007696146 | 49 | | 1 |
| apoptotic process | 0.009339407 | 95 | | 1 |
| NLS-bearing protein import into nucleus | 0.009915952 | 6 | | 1 |
